# The shared variance amongst the measures of individual differences and trait EI: a meta-meta-analytic comparison

**DOI:** 10.3389/fpsyg.2025.1635847

**Published:** 2026-01-08

**Authors:** Bogdan S. Zadorozhny, K. V. Petrides, Jinyan Yang, Dimitri van der Linden

**Affiliations:** 1 Department of Psychology, University College London (UCL), London, United Kingdom; 2 Center for Behavioural and Implementation Science, Yong Loo Lin School of Medicine, National University of Singapore, Singapore, Singapore; 3 Department of Psychology, Erasmus University Rotterdam, Rotterdam, Netherlands

**Keywords:** Big Five, creativity, differential psychology, emotional intelligence, individual differences, meta-meta-analysis, personality

## Abstract

One of the central questions in differential psychology is the extent to which its various aspects are interconnected. Past research has revealed significant correlations amongst widely different constructs including emotional intelligence, the Big Five personality traits, the Dark Triad, cognitive intelligence, and creativity, amongst others. However, few studies have investigated these interrelations concurrently with the aim of constructing a unified model. The present meta-meta-analysis was performed using exclusively meta-analytic correlational data for constructs belonging to the cognitive intelligence, trait and ability emotional intelligence (EI), personality, and miscellaneous factor categories. Relationships amongst these constructs were investigated through a series of Exploratory Factor Analyses, Exploratory Structural Equation Modeling, and Confirmatory Factor Analyses. The results indicate that the latent factor formed by the overlap between the various components of differential psychology is closely linked with trait EI and appears to represent positive psychosocial adjustment or overall well-balanced functioning of the individual.

**Systematic review registration:**

https://osf.io/8pfwq?view_only=f493ff7aa90145fca6897c258c6099c2.

## 1 Introduction

A key theme that has been explored within psychology since its inception has been the investigation of that which makes individuals differ from one another, commonly referred to as differential psychology (Ashton, 2022). This general field can be further described as comprising four categories: cognitive intelligence, emotional intelligence, personality, and assorted miscellaneous factors, which together encapsulate individual differences (Brotheridge, 2006; Cooper, 2020; Conway et al., 2021; Appelt et al., 2011).

Throughout the development of the individual differences field, two competing patterns emerged. On one hand, as certain areas of the field grew in popularity, a dizzying array of different measures emerged that were each argued by their authors to assess various aspects of a given construct. This tendency is perhaps most vividly highlighted by the sheer number of competing measures that claim to assess emotional intelligence (EI), which was estimated to be as high as 40 in a recent meta-analysis (Bru-Luna et al., 2021). On the other hand, the recurrent competing theme argues for the converse—that an assortment of various measures in a particular area of differential psychology share a common core. For instance, this phenomenon has been observed in the field of cognitive intelligence (giving rise to *g*) and in personality (producing the General Factor of Personality; GFP) (Deary et al., 2010; Rushton et al., 2008; Figueredo et al., 2004).

The correlations amongst constructs within the various categories of differential psychology have been argued to support evidence of potential general factors. However, the significant and intriguing aspect of this is that, in addition to the associations between the constructs *within* each category, significant correlations *between* these categories have also been reported in extant literature. For instance, meta-analytic estimates of the association between cognitive intelligence and ability EI has been reported to be *ρ* = 0.25 (Joseph and Newman, 2010). Cognitive ability is likewise consistently correlated with some of the Big Five personality traits, especially between cognitive ability and openness, as has been demonstrated in a large-sample study (Rammstedt et al., 2016).

Therefore, given the evidence that indicates significant relationships both within and amongst the primary aspects of differential psychology (viz., cognitive intelligence, EI, personality, and miscellaneous factors), a relevant question that arises is whether these associations may be evidence of meaningful overlap amongst them.

### 1.1 Cognitive intelligence

Perhaps the oldest and most well-known example of the trend of converging aptitudes within a domain of individual differences was the development of the general intelligence factor (*g*), as initially proposed by Spearman at the fin de siècle (turn of the 20th century), based on evidence that children’s abilities on seemingly unrelated subjects were significantly correlated (Spearman, 1904). Although the existence of *g* was historically contentious, it is presently largely undisputed, based on evidence derived from psychometric factor analysis and genetic research (Plomin and Spinath, 2002; Jensen, 1987).

On the contrary, general cognitive intelligence has also been split into different components, prominently by Cattell who divided it into fluid and crystallized intelligence (Cattell, 1963). This model was subsequently developed into what is presently known as the Cattell-Horn-Carroll theory of intelligence which includes narrow, broad, and general ability as the three respective components of intelligence (Flanagan et al., 2013). In this sense, the most accepted modern theory of cognitive intelligence is hierarchical and compatible with *g*.

Nevertheless, other researchers, including Gardner, have suggested that there are a much larger number of different intelligences, such as musical, bodily-kinesthetic, and interpersonal and intrapersonal intelligence (Visser et al., 2006; Klitgaard and Gardner, 1984). Notably, Gardner’s multiple intelligence theory remains disputed in the scientific community due to scant substantive empirical evidence that would connect it to real-life outcomes, especially relative to *g* (Gottfredson, 2003; Ferrero et al., 2021; Tuncdogan et al., 2024). Additionally, research has indicated that *g* directly and significantly loads onto many of Gardner’s multiple intelligences (Visser et al., 2006).

### 1.2 Emotional intelligence

Significantly, however, two of Gardner’s multiple intelligences (i.e., interpersonal and intrapersonal intelligence) seem to be closely tied to the construct that thereafter became known as emotional intelligence (EI) (Salovey and Mayer, 1990). EI subsequently developed into what can be regarded as the second major subsection of differential psychology and particularly gained cachet following the publication of Goleman’s eponymous book (Goleman, 1995). EI has bloomed into one of the most popular elements of contemporary psychology, enjoying particular prominence in the field of business psychology (Schmitt, 2006). As EI became increasingly predominant, the multitude of various measures that claimed to assess it generally coalesced into two major categories: ability and trait emotional intelligence (Petrides, 2011). Put succinctly, the difference between the two definitions is that whereas the former posits that emotional intelligence is an aptitude best assessed through performance tests, the latter regards EI as a set of personality-like dispositions that are best measured through self-evaluation (Petrides, 2011). Typically, correlations between the two have been reported to be modest, e.g., a corrected meta-analytic correlation of *ρ* = 0.26 (Joseph and Newman, 2010).

### 1.3 Personality

The third major subsection of differential psychology is perhaps the widest and most disparate and can be broadly described as personality (Furnham and Kanazawa, 2020). One of the earliest researchers that suggested a multifactorial system of personality was the eminent psychologist, Hans Eysenck, who proposed a bifactorial model that was later expanded to three dimensions, viz., extraversion, neuroticism, and psychoticism (van Kampen, 2009). Eysenck’s three-factor model was in significant contention with a competing five-factor model that was advocated by Costa and McCrae (1995). Interestingly, both camps of researchers argued that one another’s model could be reduced to the respective number of factors of which their preferred model was composed—a fact that could be construed as calling into question the factorial nature of both models. However, one of the models of personality that gained the most popularity was the Big Five model, which is composed of Openness, Conscientiousness, Extraversion, Agreeableness, and Neuroticism (Vassend and Skrondal, 2011; Anglim et al., 2022), although a significant number of competing models had also been developed.

Intriguingly, and perhaps predictably, a debate similar to the one that unfolded surrounding cognitive intelligence erupted in the domain of personality. While models of personality were composed of varying numbers of dimensions as initially proposed, evidence emerged that suggested that these dimensions were not fundamental, but in fact showed consistent intercorrelations, as demonstrated by theoretical reviews (Digman, 1997; Hofstee, 2003; Rushton and Irwing, 2009), single empirical studies (e.g., Musek, 2007), and meta-analytic evidence (van der Linden et al., 2010). This led to the positing of higher-order factors of personality, such as the two-factor model consisting of stability and plasticity (van der Linden et al., 2017). Furthermore, van der Linden et al. (2017) presented a model of personality wherein additionally to the Big Five traits loading onto stability and plasticity, the latter two factors further load onto an apex factor called the General Factor of Personality (GFP). Interestingly, the GFP had been meta-analytically found to be very strongly correlated (*r* = 0.86) with trait emotional intelligence (trait EI) (van der Linden et al., 2017), which led the authors to suggest that the two constructs may in fact be synonymous.

Outside of general personality assessment, instruments have emerged that focus on explicitly assessing the aversive aspects of personality, prominently including narcissism, psychopathy, and Machiavellianism, which are presently combined in models such as the Dark Triad and, in combination, with sadism, the dark tetrad (Dinić et al., 2021; Bonfá-Araujo et al., 2022). Narcissism is characterized by a disposition toward self-absorption, grandiosity, and is often associated with low empathy (di Giacomo et al., 2023). Psychopathy refers to an assortment of antisocial tendencies including boldness, social dominance, meanness, disinhibition, and is likewise associated with low empathy (Patrick, 2022; De Brito et al., 2021). Unlike the prior two traits, Machiavellianism is associated positively with empathy, but Machiavellians harness their empathic and interpersonal abilities cynically and exploitatively to achieve selfish aims (Jones and Mueller, 2021). Finally, sadism is defined as the tendency to behave cruelly toward others and especially by the desire to intentionally inflict pain (Foulkes, 2019).

Significant positive correlations have been found amongst the dark traits and emotional intelligence, despite the fact that EI is understood to be a positive rather than a maladaptive aspect of personality. In particular, recent meta-analyses have repeatedly found EI to be positively associated with narcissism (Michels and Schulze, 2021; Nguyen et al., 2022). A proposed theory explains this relationship through reference to a purported dark side of trait emotional intelligence (Davis and Nichols, 2016).

### 1.4 Miscellaneous measures

Finally, the fourth subsection of differential psychology consists of an assortment of miscellaneous integrative and contextual factors that do not neatly fit into the aforementioned categories of cognitive intelligence, emotional intelligence, and personality, but that do have significant associations with constructs belonging to said categories. These are primarily performance, outcome, or indicator measures. Amongst them are academic achievement; career-related factors: job performance, job satisfaction, leadership, and cultural intelligence (CQ); creative and aesthetic measures: creative achievement, creativity, and general aesthetic taste (T); wellbeing and related measures: self-concept, self-esteem, general self-efficacy, and satisfaction with life; as well as spirituality/religiosity. Of these different categories of measures, the data obtained throughout the meta-meta-analytic process provided linkages with the primary three categories only in the case of creativity.

Creativity is broadly defined as the ability to come up with novel and original solutions to a particular task (Zare and Flinchbaugh, 2018). Creativity has been found to be significantly correlated with cognitive ability, with one second-order meta-analytic estimate suggesting a relationship of *r* = 0.17 (Da Costa et al., 2015). However, correlations amongst these two constructs have been found to vary widely dependent on how creativity and intelligence are defined respective to one another and the type of creativity assessed (e.g., creative potential vs. outcome; Plucker et al., 2015, Runco, 2022).

Creativity and emotional intelligence are also reported to be positively related—though meta-analytic evidence has indicated that the relationship of creativity with trait EI is significantly stronger than the relationship between creativity and ability EI (*r* = 0.35 vs. *r* = 0.08; Xu et al., 2019).

Finally, with respect to the relationship between measures of creativity and the Big Five, meta-analytic data has indicated that creativity is most strongly linked to openness (*r* = 0.47), followed by extraversion (*r* = 0.26) and conscientiousness (*r* = 0.13; Karwowski and Lebuda, 2015).

In summary, while a number of miscellaneous performance, outcome, and contextual measures have been linked to the major domains of individual differences, amongst them creativity emerges as a linking construct. Namely, creativity has been found to be positively correlated with both cognitive ability and trait emotional intelligence as well as with personality measures. This therefore indicates that creativity may serve as a bridge across the aforementioned domains of individual differences and thereby is the miscellaneous measure that most merits special attention.

### 1.5 Present study

The general aim of this study was to identify whether a single latent factor can account for the shared variance amongst a broad assortment of differential psychology measures (including cognitive intelligence, emotional intelligence, personality, and miscellaneous constructs) by means of meta-analytic correlational data.

Conducting data analysis on meta-analytic data poses certain unique challenges that necessitate discussion. The process of fitting Structural Equation Models (SEM) to meta-analytic data is referred to as MASEM, or meta-analytic structural equation modeling (Jak et al., 2021). One of the advantages of the MASEM approach is that it empowers researchers to extend beyond the scope of the standard meta-analysis which is generally restricted to investigation of a single bivariate relationship (Oh, 2020). Another benefit is that it enables researchers to synthesize a complete correlational matrix, even if no single included meta-analysis includes relationships amongst all variables of interest (Oh, 2020).

The general procedure of conducting a MASEM consists of two steps wherein 1) a correlational matrix is constructed based on meta-analytic correlations derived from prior meta-analyses and 2) said input matrix is then utilized to carry out subsequent analyses (Oh, 2020; Cheung, 2014). Furthermore, there are two approaches to how the analyses are conducted: fixed- and random-effects models (Cheung, 2014; Sheng et al., 2016). The former family of approaches only deals with point estimates of effect sizes while ignoring the variances around them, leading to SEM results that unpredictably both under- or overestimate parameters (Oh, 2020; Jak and Cheung, 2022). The alternative option is the use of the two-stage SEM (TSSEM) method (Sheng et al., 2016) that allows for the unambiguous determination of sample size, while simultaneously accounting for sampling variance between meta-analyses by weighing the primary studies appropriately, i.e., by giving less weight to studies with greater amounts of sampling variance (Cheung and Chan, 2005).

However, TSSEM requires researchers to have access to the data of all primary studies that are included within each meta-analysis incorporated in the investigation (Oh, 2020). In instances where this is considered impossible or impractical, the full-information MASEM (FIMASEM) or the traditional SEM approaches are suggested (Oh, 2020). In the absence of standard deviation data, an option is to populate the input correlational matrix with previously derived meta-analytic estimates along with some corresponding sample size (Oh, 2020). However, such an approach may not sufficiently account for effect size heterogeneity (Jak and Cheung, 2022).

Considering the large number of meta-analyses that were incorporated in the present study, it was determined to proceed with SEM analyses using a correlational matrix composed of meta-analytic estimates as input.

Given extant meta-analytic evidence that indicates significant relationships amongst pairs of constructs belonging to the four enumerated primary aspects of differential psychology, the present meta-meta-analysis was designed to holistically examine the pattern of correlations amongst measures belonging to the aforementioned categories. Meta-analyses were included if they reported on the correlations between at least two constructs under investigation. In instances where correlational data for construct pairs was not obtained, the affected constructs were dropped from analysis.

The primary goal of the current study was to establish whether the relationships amongst differential psychology constructs form a latent construct that is strongly related to trait EI. It was expected that a latent factor would be obtained with observed uneven construct loadings. The identification of such a factor, however, even if it accounted for a small percentage of variance, would support the presence of a common thread underlying diverse constructs in differential psychology. This, in turn, could represent a psychological tendency that influences behavior across all domains. The breadth of constructs investigated in this research will allow us to conduct a preliminary examination of the hypothesis that a single background variable underpins mental activity in its entirety (Petrides, 2021).

Statistical methods, primarily designed to study variation and difference, are often ill-suited for the discovery of a quality that is omnipresent and equally present in mental activity. Nevertheless, any evidence of commonality across the diverse constructs examined herein would support this hypothesis. Such analyses would also contribute to the broader discussion of the role and nature of general factors in differential psychology.

## 2 Method

### 2.1 Transparency and openness

In accordance with meta-analytic best practices, a protocol was prepared according to PRISMA-P guidelines (Shamseer et al., 2015) and registered on Open Science Framework (OSF).^1^ There were deviations from the registered protocol, each of which had a minimal impact on the conclusions for the following reasons: a) Mini meta-analyses were not performed to avoid introducing bias from primary studies, and instead, only published meta-analyses were used to maximize data quality and homogeneity; b) Quality assessments were omitted because all included studies were published meta-analyses, for which standard primary-study quality ratings are not appropriate, while the meta-analytic process itself screens out low-quality research; c) When multiple meta-analyses existed for a construct pair, recency was prioritized over sample size to best reflect the current state of the literature; d) Fisher’s *z*-transformed matrices could not be analyzed due to non-positive definite matrices, so Pearson correlations were used instead, which is a standard approach in meta-analytic SEM; e) FE/RE TSSEM and OSMASEM methods were not used due to insufficient overlap amongst studies, so a pooled fixed-effects meta-analytic correlation matrix was adopted as a recognized alternative; f) Models including subfactors did not converge, which itself is informative and does not bias the main finding of a latent factor; g) Though sensitivity analyses were not performed, robustness was checked by means of alternative model specifications. In all cases, analytic choices were transparently data-driven and aimed at maximizing the validity and interpretability of the results.

### 2.2 Preparation (pre-pilot stage)

As this was an exploratory research project, it was necessary for the authors to first prepare a list of keywords to target for inclusion prior to initiating the meta-meta-analysis. Therefore, 24 articles that were previously known to the authors were examined in order to generate a list of keywords that would be utilized to search for a general factor within the domain of differential psychology (see Supplementary Table S1). Thereafter, this initial list was expanded by including logical extensions and the resultant 120 keywords were sorted into five categories on the basis of their general categorical themes: namely, cognitive intelligence, emotional intelligence, personality, general factors, and miscellaneous.

Following the construction of this initial keyword list, test searches were conducted to assess the viability of the search strategy, with select meta-analyses retrieved and inspected to identify the constructs and measures they included. These constructs were subsequently combined and categorized, for a total of 24 construct categories that included 143 base constructs. These categories were: Overall Emotional Intelligence; Academic Achievement; Motivation; Empathy; General Factor of Personality; Cognitive Intelligence; Antisocial Personality Disorder; General Dark Triad; Cultural Intelligence; Generic Performance; General Creative Achievement; Defensiveness; Divergent Thinking; Career-Related Factors; General Taste; Leadership; Self-Concept; Self-Efficacy; Values; Interpersonal Factors; Response Time; WellBeing; and Religiosity. Following a literature review, this initial list was subsequently pared down to 19 categories.

Using this as foundation, an updated list of search keywords was created, with new keywords added while some older keywords were truncated. Simultaneously, a list of 34 target constructs was developed by means of author consensus on the basis of the aforementioned searches in order to precisely enumerate the constructs between which correlations would be subsequently sought and extracted. The list of targeted constructs was as follows: Ability Emotional Intelligence; Trait Emotional Intelligence; Emotional Manipulation (EMS); Academic Achievement (including Academic Performance and Academic Success); Affective Empathy; Cognitive Empathy; Openness; Conscientiousness; Extraversion; Agreeableness; Neuroticism / Emotional Stability; Honesty-Humility; Eysenckian Lie scale; Eysenckian Psychoticism; Full-Scale IQ / General Mental Ability; Crystallized Intelligence; Fluid Intelligence; Antisocial Personality Disorder (including Antisocial Behavior); Machiavellianism; Narcissism; Psychopathy; Cultural Intelligence (CQ); Job Performance; Job Satisfaction; Creative Achievement; Creativity; General Aesthetic Taste (T); Leadership; Self-Concept / Self-Esteem (including General Self-Concept, Self-Perceptions, and Self-Esteem); Self-Efficacy; General Values; General WellBeing (including WellBeing (various types) and SWB (Subjective WellBeing)); Satisfaction with Life; and Spirituality/Religiosity.

Following the development of the updated keyword lists (containing a total of 138 keywords; see Supplementary Table S2), a series of pilot searches were conducted to validate the search algorithm. This was done by checking whether certain meta-analyses known to the authors as relevant and related to the research question were appearing in the results as expected. Additionally, a small sample of the papers (*n* = 5) was examined to determine whether the obtained studies contained the required data. Given the fact that certain meta-analyses previously known to the authors failed to appear in the results, the search algorithm was consequently amended. Specifically, rather than conducting searches within the author-specified keywords specifically, searches were conducted within all available fields instead, including paper abstracts.

As the present study was exploratory in nature, the preparatory stage was conducted in a systematic and iterative manner so as to identify and categorize the relevant constructs for inclusion in the meta-meta-analysis. First, preliminary keywords were collated from an initial collection of 24 articles. This group of keywords was then expanded to 120 terms across thematic categories. Following test searches and a literature review, 19 categories of interest were developed, on the basis of which 34 constructs were targeted. Once the keyword lists were compiled, pilot searches were conducted to validate the search strategy. The algorithm was subsequently adjusted to search all fields to ensure all relevant articles were retrieved. In the end, a comprehensive and validated set of constructs and keywords was developed, which lay the foundation for the proceeding phases of the study.

### 2.3 Literature search

The present meta-meta-analysis sought to identify all English-language studies that reported at least one meta-analytic correlation between two or more targeted constructs. The primary set of searches was conducted in January 2024 utilizing a search algorithm on Web of Science that included all studies published through to December 31st, 2022. A publication date cutoff was incorporated to enable interrater replicability. Due to the large number of articles that were expected to be retrieved, no search expansion approach was utilized. Corresponding authors were not contacted as the research was designed to not incorporate any primary or non-publicly-accessible data to ensure replicability and extension. The searches were repeated in June 2024 to update the scope of the meta-meta-analysis.

The search keywords included all possible combinations from Supplementary Table S2, along with the term “meta-analysis,” targeting all text fields. Article titles and keywords were collected for all results. In addition to the studies retrieved using the primary approach, manual searches were conducted across other databases (Ovid PsycArticles, Wiley Online Library, Elsevier ScienceDirect, Medline/Pubmed, Taylor and Francis, Google Scholar, and ResearchGate, in that respective order) for construct pairs lacking correlational data or when the sample size was considered insufficient. In all, two additional meta-analyses were obtained and retained using this secondary methodology.

### 2.4 Screening

**Figure 1:**
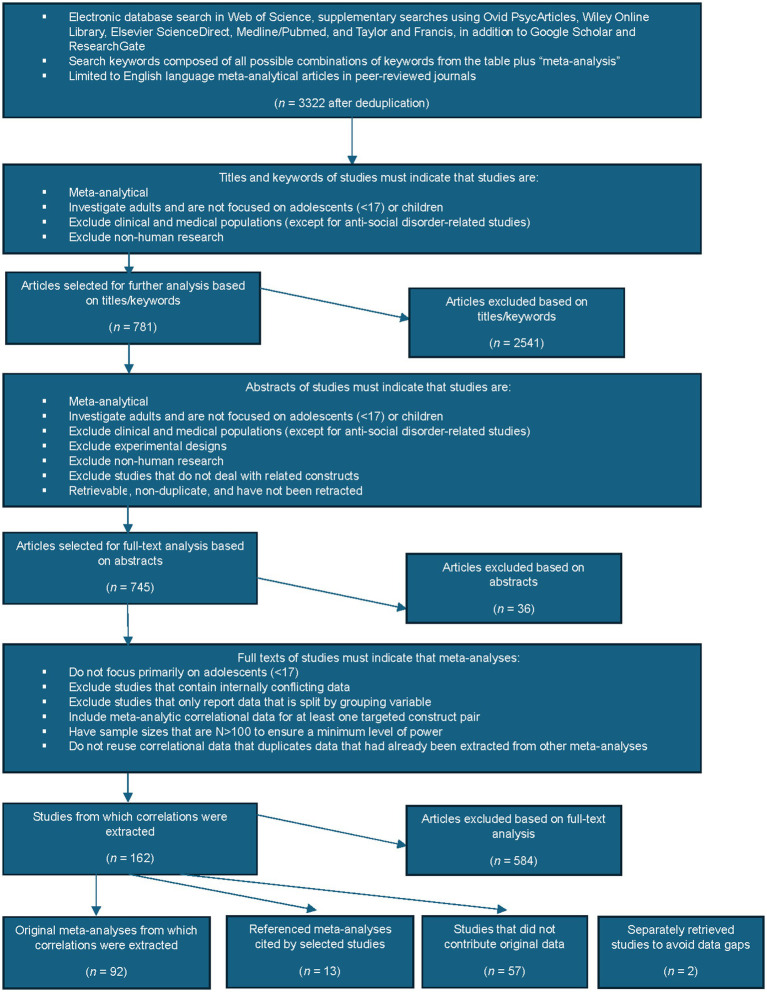
Flow diagram representing the literature screening process.

Figure 1 illustrates the flow of articles through the screening stages. The number of studies that were initially retrieved was 3,322 following deduplication. Next, the papers were screened in three main phases (keyword/title, abstract, and full text screening). It was expected based on the pilot results, that the title and keyword screening phase would exclude a significant quantity of papers and was deemed necessary due to the high number of papers that were expected to be retrieved. The remaining stages of the screening process were standard and were inspired by past meta-analyses that were conducted in this area (Bücker et al., 2018; Fong et al., 2021; Gesel et al., 2020).

The papers were first screened based on their titles and author keywords. To minimize the risk of bias, ISSN/DOI data, publication dates, and journal names were hidden in this phase. The criteria were that the papers had to be meta-analytical and not focused on children and/or adolescents (<17 in age). Additionally, studies conducted on clinical populations were excluded except for when the construct under investigation was Anti-Social Disorder (ASD). Non-human research was also excluded. Following this initial screening phase, 781 studies were retained while 2,541 studies were excluded. Of the 2,541 studies that were excluded, the top reasons for exclusion were: 1,357 papers were excluded as they did not appear to involve related concepts, 607 were removed because they were conducted on clinical populations, and 237 because they were conducted on either children or adolescents.

Next, the 781 retained papers from the prior step were screened based on their abstracts according to the same criteria, but also excluding experimental designs, studies that did not include related constructs, and excluding studies that were unretrievable, duplicates, or retracted. A further 36 studies were excluded during this stage, with 745 studies retained for full-text analysis.

The full text screening phase excluded meta-analyses that incorporated primary studies focused primarily on adolescents (<17), contained internally inconsistent data, were missing summary correlational data, did not contain relevant data, or had sample sizes that were *N* < 100. The majority of the 584 articles excluded at the full text screening phase were excluded for the following reasons, in descending order: 365 did not contain relevant data, 78 did not contain summary level data (they included only data broken by category, for instance, only by gender), 71 were not true meta-analyses (for instance, were internal meta-analyses), and 43 were conducted on adolescents/children. Correlations were extracted from 162 studies.

The search repetition, conducted separately at a later date, yielded a further 70 studies, of which 26 were retained on the basis of title/keyword screening, of which 10 were kept following abstract screening. Full-text analysis revealed that one of these papers contained a relevant correlation.

### 2.5 Data extraction and verification

In the first phase, 162 studies were found to contain relevant data, of which 92 contained meta-analytic correlations that were directly extracted, 13 referred to prior meta-analyses from which data was extracted, and 57 did not contain novel meta-analytic correlational data (viz., said data was already available in other cited meta-analyses). One additional correlation was extracted from the search repetition and two studies were retrieved separately thereafter in order to avoid data gaps. In total, there were 404 samples extracted from 108 articles (see Supplementary Table S3). For each study, the following data was extracted: construct pairing, correlational value, correlation type, *N*, *k*, uncorrected mean correlations, corrected correlations (rho), data source information (original meta-analysis vs. cited meta-analysis), and metadata (author names, year of publication, article title). In addition, a list of measures that were used to operationalize all constructs was extracted from each included meta-analysis, concatenated, and is presented in Supplementary Table S4. For the narcissism construct, all data were derived from instruments that specifically assess grandiose narcissism (and not vulnerable narcissism). Therefore, the narcissism indicator in the current study should be interpreted as representing grandiose narcissism only. For the Big Five intercorrelations specifically, the decision was made to reuse correlational data specified in a prior meta-analysis, as the sample size was substantial (van der Linden et al., 2010).

The secondary researcher performed a duplication of the searches, with the goal of confirming that the same studies were retrieved. Next, they screened the articles based on the agreed-upon inclusion/exclusion criteria in order to reduce potential selection bias. In all phases, all discrepancies were discussed and resolved prior to moving on to the next stage. Researcher interrater agreement was assessed by means of Cohen’s kappa and was calculated to be 0.94, which was interpreted as nearly perfect agreement.

Data from the included studies were manually extracted and assembled in a 34 × 34 matrix and inspected to assess what correlational data was found and what construct combinations were missing data. All extracted correlations were either corrected or uncorrected Pearson correlations. Given that the resultant matrix contained gaps, the matrix was pared down to 12 × 12, including data from the additional meta-analyses. The secondary researcher also extracted correlational data from all studies and these data extractions were cross verified to ensure no discrepancies were present. Appendix Table A5 specifies which studies were utilized for the construction of this matrix.

### 2.6 Data analysis plan

Statistical analyses were performed using SPSS v. 29, SPSS AMOS v. 29, and RStudio v. 2023.12.0 + 369. The general statistical procedure that was followed adhered to the recommendations in prior literature (Morin et al., 2020; Morin, 2023). The primary goal of the present study was to assess whether a general latent factor could account for the shared variance amongst a range of differential psychology constructs using meta-analytic data. In pursuit of this aim, we employed a stepwise analytic strategy, progressing from exploratory to increasingly more constrained models.

Specifically, we conducted 1) Exploratory Factor Analyses (EFA); 2) Exploratory Structure Equation Modeling (ESEM) within a Confirmatory Factor Analysis (ESEM-within-CFA) (Morin et al., 2013); 3) a series of Confirmatory Factor Analyses (CFA); 4) bifactor CFA and ESEM models; 5) and finally a unifactorial CFA solution with constraints.

EFA was conducted first in an effort to identify the underlying factor structure amongst the included constructs without any theory-derived constraints. This stage was necessary due to the breadth of the included constructs and the resultant lack of confidence in any concrete *a priori* model. Next, ESEM-within-CFA was used to model cross-loadings utilizing a more flexible representation that is afforded by this approach as compared to standard CFA. Following this, CFA was employed to assess whether a multifactorial or a unifactorial model better accounted for the observed pattern of correlations. In order to examine the possibility of a solution that included both a latent general factor as well as a number of domain specific factors, bifactor models were assessed. Finally, a unifactorial CFA model was used to determine if model fit could be improved by means of accounting for a number of known construct interrelations that are not captured by the latent factor in isolation. At each stage, fit indices were used to guide decisions, favoring models that were both more theoretically plausible and better fitting. By following this sequence, we were able to test alternative explanations that were driven by both data and theory and to determine whether the hypothesized latent factor could explain the observed shared variance amongst the constructs.

Prior to initiating data analysis, an input matrix was generated that included all constructs for which meta-analytic correlations were obtained. However, seeing as this matrix included gaps, the decision was made to remove the constructs for which meta-analytic estimates were not obtained. This approach was justified by the concern that with such a great number of empty cells, there was a significant risk of bias and Type I error (Sheng et al., 2016). In instances where both reliability-uncorrected and corrected correlations were provided, the corrected values were preferred. In addition, the possibility of overlapping primary studies was considered if multiple retrieved meta-analyses were allowed to contribute correlational data for the same pair of constructs (Türk et al., 2023). To avoid this issue, for each unique construct pair, only the meta-analysis with the largest sample size was retained. The resultant gap-free construct matrix is presented in Table 1.

**Table 1:** Gapless pooled matrix of meta-analytic correlations.

Measure	1	2	3	4	5	6	7	8	9	10	11	12
1. Openness	–											
2. Conscientiousness	0.20	–										
3. Extraversion	0.43	0.29	–									
4. Agreeableness	0.21	0.43	0.26	–								
5. Neuroticism	−0.17	−0.43	−0.36	−0.36	–							
6. Ability EI	0.18	0.11	0.06	0.20	−0.11	–						
7. Trait EI	0.38	0.47	0.56	0.45	−0.68	0.26	–					
8. Cognitive ability	0.17	−0.03	−0.02	0.00	−0.07	0.25	0.06	–				
9. Machiavellianism	−0.02	−0.19	−0.02	−0.40	0.13	−0.31	−0.29	0.03**	–			
10. Narcissism	0.25	0.11	0.49	−0.36	−0.20	−0.16	0.18	0.01**	0.34	–		
11. Psychopathy	−0.03	−0.24	0.01	−0.47	0.08	−0.23	−0.25	−0.06	0.58	0.37	–	
12. Creativity	0.47	0.13	0.18	0.07	−0.12	0.08	0.35	0.33**	0.06	0.15	0.03	–

*k* = 22; harmonic mean *N* = 10,319.

^**^ Marked values derived from meta-analyses that were retrieved separately to the main searches.

With respect to the sample size specified for analysis, we followed the best practice that recommended using the harmonic mean as the sample size for the model specification as the most defensible choice from the standpoint of statistical rigor (Sheng et al., 2016). Finally, in accordance with the recommendations in the same article, we checked whether the input matrix was nonpositive definite prior to proceeding with analysis.

The goodness of fit of the various models assessed was carried out through the use of the Comparative Fit Index (CFI), the Tucker-Lewis Index (TLI), the Root Mean Square Error of Approximation (RMSEA) (including 90% confidence intervals), and the Standardized Root Mean Square Residual (SRMR). The fit thresholds that indicated good fit were selected from prior literature as follows: *CFI* > 0.90, *TLI* > 0.90, *RMSEA* < 0.08, *SRMR* < 0.08 (Hoyle, 1995).

## 3 Results

As an initial step, we used the meta-analytic correlation matrix (Table 1) as input for Exploratory Factor Analysis (EFA) to determine how many factors would be suggested. EFA was conducted by approximating the number of purported factors through Eigenvalue estimation, wherein factors with an Eigenvalue of greater than 1 were retained, using the Promax rotation and Principal Axis Factoring. Analysis of the results indicated that a three-factor solution (Table 2) explained the greatest amount of variance at 47%. The three factors seemed to mainly represent 1) conscientiousness, extraversion, neuroticism, and trait EI, 2) disagreeableness/the dark core, and 3) openness/cognitive ability/creativity, respectively.

**Table 2:** Factor pattern matrix.

Measure	Factor 1	Factor 2	Factor 3
1. Openness	0.31	0.08	**0.49**
2. Conscientiousness	**0.55**	−0.19	−0.09
3. Extraversion	**0.72**	0.21	0.03
4. Agreeableness	0.38	**−0.59**	−0.05
5. Neuroticism	**−0.68**	0.06	0.08
6. Ability EI	0.01	−0.34	0.26
7. Trait EI	***0.80***	−0.18	0.11
8. Cognitive ability	−0.16	−0.05	**0.53**
9. Machiavellianism	−0.10	**0.66**	0.07
10. Narcissism	0.49	**0.72**	0.04
11. Psychopathy	−0.05	***0.70***	0.02
12. Creativity	0.08	0.10	***0.68***

Values that are both bold and italicized indicate selected referents, while bolded items represent indicators.

Further tests, however, suggested that the three aforementioned factors were non-oblique, which points to the possibility of commonality amongst them. Specifically, an ESEM-within-CFA (Silvestrin and de Beer, 2022) based on the independent three factor model failed to converge, while a targeted rotation ESEM yielded a non-positive-definite matrix. Similarly, a three-factor Confirmatory Factor Analysis (CFA) exhibited non-positive variances. Following Swami et al. (2023), bifactorial analyses (including CFA and ESEM) were then performed. The bifactorial CFA based on the three-factor EFA demonstrated a slightly improved fit but remained unsatisfactory, while the bifactor ESEM model failed to converge.

**Figure 2:**
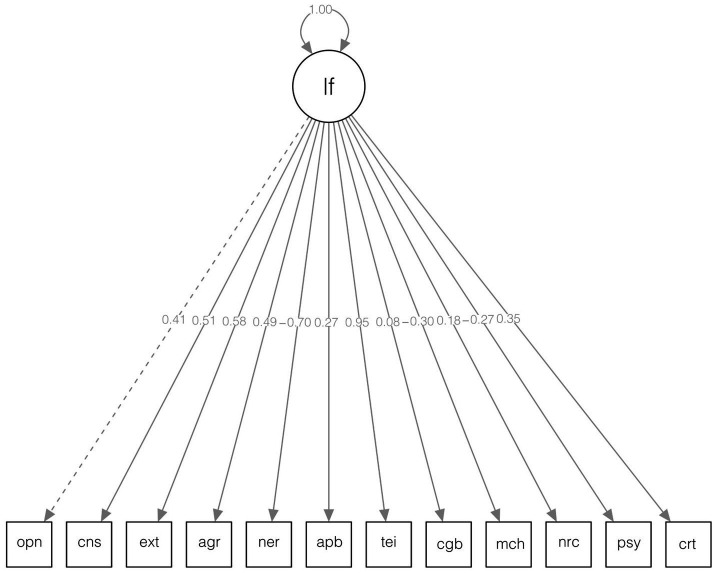
Original unifactorial CFA model diagram. The indicated numerical values toward the center of the diagram correspond to the standardized factor loadings of each indicator. lf, latent factor; opn, openness; cns, conscientiousness; ext, extraversion; agr, agreeableness; ner, neuroticism; apb, ability EI (performance-based); tei, trait EI; cgb, cognitive ability; mch, Machiavellianism; nrc, narcissism; psy, psychopathy; crt, creativity.

We then moved to testing unifactorial models that were accordant with the key hypothesis of the study. We first constructed a unifactorial CFA with all constructs loading onto a single general factor, which resulted in a poor-fitting model (Figure 2), *χ*^2^(54) = 21643.44, *p* < 0.001; *CFI* = 0.469; *TLI* = 0.352; *RMSEA* = 0.206, 90% CI [0.204, 0.209]; *SRMR* = 0.154. Appendix Tables A2, A3 contain the unstandardized loadings and the error variances for this model. The primary reason for this was the presence of intercorrelations between the unique aspects of the measures. This is logical, as it would not be viable to expect that all interrelations would be captured by a single factor.

**Figure 3:**
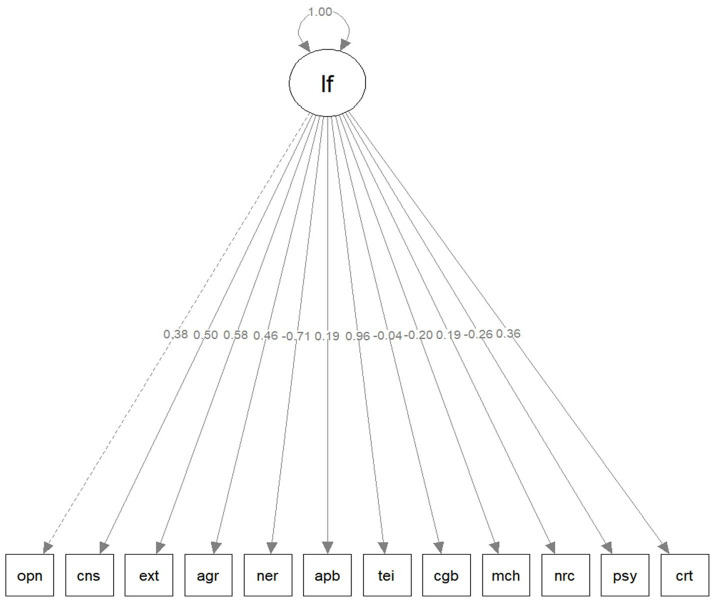
Modified unifactorial CFA model diagram. The indicated numerical values toward the center of the diagram correspond to the standardized factor loadings of each indicator. lf, latent factor; opn, openness; cns, conscientiousness; ext, extraversion; agr, agreeableness; ner, neuroticism; apb, ability EI (performance-based); tei, trait EI; cgb, cognitive ability; mch, Machiavellianism; nrc, narcissism; psy, psychopathy; crt, creativity.

Therefore, we proceeded with an exploratory analysis wherein a modified model was fitted (Figure 3) that incorporated the same single latent general factor along with 33 covariances amongst the indicator variables. The covariances were modeled so as to better reflect the data as completely independent unique variance was considered implausible. The specific modeled covariances are included in Table 3. The resultant fit of the model was good, *χ*^2^(21) = 1241.56, *p* < 0.001; *CFI* = 0.97; *TLI* = 0.91; *RMSEA* = 0.079, 90% CI [0.075, 0.082]; *SRMR* = 0.040.

**Table 3:** Modeled covariances between the unique variances (non-general factor).

Construct	1	2	3	4	5	6	7	8	9	10	11	12
1. Openness	–											
2. Conscientiousness		–										
3. Extraversion	X		–									
4. Agreeableness	X	X		–								
5. Neuroticism					–							
6. Ability EI	X					–						
7. Trait EI						X	–					
8. Cognitive ability	X			X	X	X	X	–				
9. Machiavellianism			X	X		X	X		–			
10. Narcissism	X		X	X	X	X			X	–		
11. Psychopathy			X		X	X		X	X	X	–	
12. Creativity	X		X	X	X			X	X		X	–

Figure 3 illustrates a simplified version of the model. It indicates that the majority of variables had moderate loadings (ranging from |0.19| to |0.46|), whereas a few variables showed substantial loadings (ranging from |0.50| to |0.96|).

## 4 Discussion

There is a great breadth of studies that examine one or more of the correlational relationships between constructs belonging to the various categories of differential psychology, such as between cognitive ability and ability emotional intelligence (ability EI; Joseph and Newman, 2010). However, despite the meta-analytic evidence that supports the theory that constructs belonging to the personality, cognitive ability, emotional intelligence, and other domains are intercorrelated to some extent, no meta-analyses known to the authors have assessed these relationships holistically from the standpoint of exploring whether there is overlap amongst the differential psychology constructs. Therefore, the present meta-meta-analysis was designed to examine whether the relationships amongst constructs belonging to these various categories provide evidence of not just links between specific pairs of constructs, as established in extant literature, but are rather, taken together, plausibly indicative of the existence of a construct that plays a connecting role in differential psychology.

Utilizing a matrix composed of meta-analytic correlations as input, a number of approaches were employed to assess the evidence for such a factor, starting with Exploratory Factor Analysis (EFA), which was justified by the fact that there was no specific pre-existing evidence that would strongly indicate a particular factorial structure (Marsh et al., 2014). EFA results initially suggested a three-factor solution and therefore this was then utilized to inform the development of subsequent models. Crucially, however, these models either failed to converge or contained non-positive variances.

When non-positive variance is obtained within a statistical model, this implies that some of its factors are potentially non-redundant (Sajtos et al., 2021). Thus, it was decided to proceed with a model that consisted of just one factor, which converged, although it demonstrated poor fit. Subsequently, a modified version of this model was developed by means of inspection of its modification indices. These revealed a number of covariances and mixed loadings that were not unexpected due to their presence in extant literature. For example, the unique variance (relative to the latent factor) of extraversion correlated (*r* = 0.37) with the unique variance of narcissism, and the unique variance of openness correlated (*r* = 0.30) with creativity. In other words, known relationships between indicator variables, as they were not explicitly incorporated within the initial unifactorial model, reduced its overall fit.

When a model was constructed that incorporated some of these covariances, however, good fit was achieved, which suggests that a latent factor does emerge amongst emotional intelligence, personality, and associated measures, although it does not load onto all measures equally and notably cognitive ability appears to fall largely outside of it. This thereby indicates that although the obtained latent factor may not fully explain the overall variance amongst these constructs, it is nevertheless relevant to the structure of their relationships. However, it should be noted that the loadings of the latent factor onto the individual items did not significantly change between the models with and without the covariances, suggesting support for the validity of the latent factor.

These results, analogously to the findings with respect to *g* and the General Factor of Personality (GFP), provide initial evidence for the notion that there may be a common thread underpinning the different constructs that belong to the various domains of differential psychology (Tuncdogan et al., 2024). Notably, the GFP itself has been reported to be related to a wide array of psychological constructs, including wellbeing, mental health, self-esteem, and motivation (Musek, 2017). Therefore, it is plausible that the latent factor suggested in the present study may exhibit some overlap with the GFP.

Alternatively, the present latent factor may be representative of a central psychological tendency that has an impact on all aspects of individual behavior. Furthermore, despite differences in individual behavior that are derived from environmental, contextual, and cultural factors (Bouchard, 1993), it is plausible that a general factor perspective would allow for the construction of a universal holistic model that would encapsulate the full behavioral range of an individual that is obscured when behavior is analyzed on the level of disconnected constructs, regarded solely in isolation from one another.

Exploring the obtained results in further detail, the strength of the loading of the latent factor onto the indicator measures varied greatly from construct to construct, as was hypothesized. Of these, by far the greatest was that of trait emotional intelligence (trait EI) (0.96). Considering the strength of the loading, trait EI emerges as the closest observed proxy for the shared variance amongst the differential psychology measures included in the present study.

In other words, trait EI may play a unique role as the connecting link between the various components of individual differences. This may mean that trait EI activates combinations of differential psychology components, the interaction between which is necessary for achieving desired outcomes in a range of situations. This accords with the results of prior studies that indicated that trait EI plays a predictive role in association with a wide gamut of life outcomes, including lower incidence of harmful behaviors such as self-harm and higher incidence of health-promoting behaviors; academic and job performance; higher relationship satisfaction; and favorable parenting practices (Petrides et al., 2016; Parker et al., 2021). Therefore, trait EI represents overall positively adjusted global functioning, comprising aspects of human inter- and intra-relational activity (Petrides, 2011). Accordingly, trait EI has also been found to be correlated with general wellbeing and happiness (Andrei et al., 2014) and to be negatively associated with anxiety and mood disorders (Mikolajczak et al., 2007).

Therefore, the latent factor can be interpreted as representing positive psychosocial adjustment or well-balanced functioning. This invites comparison with the construct of resilience which is defined as the capacity to recover and adapt to threats to functioning or development and to change one’s reactions to meet the dynamic situational demands of life (Oshio et al., 2018). Meta-analytic research has indicated that resilience is strongly associated with higher levels of conscientiousness, extraversion, agreeableness, and openness, and lower levels of neuroticism which closely mirrors the loading structure observed in our results (Oshio et al., 2018).

On a related note, neuroticism (−0.71) is the next strongest loading of the latent factor. As high levels of neuroticism are connected with the incidence of mental disorders, this thereby implies that the latent factor may represent an overall high level of psychological functioning (Ormel et al., 2013). Neuroticism is also linked with a host of negative life outcomes including emotional dysregulation, divorce, and early mortality (Mroczek and Spiro, 2007; Kokkonen and Pulkkinen, 2001). Thereby, it can be hypothesized that the presently suggested latent factor is thus linked to the presence of positive or at least the absence of negative life outcomes.

Following trait EI and neuroticism, the latent factor loaded moderately onto extraversion (0.58), conscientiousness (0.50), and agreeableness (0.46). The strong loading onto extraversion supports the association of the latent factor with positive social functioning and underscores extraversion’s importance in overall wellbeing, which is well-supported in extant literature (Sun et al., 2020). The relationship with conscientiousness was also moderate and this construct has been found to be correlated with job performance and career success, over and above the other Big Five traits (Barrick et al., 2001). It is also the Big Five trait that is most strongly correlated with subjective wellbeing and life satisfaction (Steel et al., 2008). The loading onto agreeableness was just slightly less strong than onto conscientiousness and this construct is strongly negatively associated with the aversive aspects of personality, including psychopathy, narcissism, and Machiavellianism (Moshagen et al., 2018). On the other hand, high levels of agreeableness are strongly correlated with empathy, with one study reporting a relationship of *r* = 0.75, even suggesting that the two constructs may be synonymous (Nettle, 2007). Thus, this implies that the present latent factor may be linked with achievement drive, wellbeing, satisfaction with life, and empathic feelings and behaviors.

Next, the latent factor loaded moderately onto both openness (0.38) and creativity (0.36). These two measures have been found to be rather strongly associated (*r* = 0.37–0.42) with one another in extant literature (McCrae, 1987). This is perhaps unsurprising as creativity, defined as divergent thinking, is quite close to the definition of openness. Therefore, part of the latent factor represents orientation toward novel ways of thinking, behaving, and creative disposition. Additionally, both openness and creativity are strongly correlated with cognitive ability (Sternberg, 1985; McCrae, 1987).

Conversely, the latent factor had a small and negative loading onto cognitive ability (−0.04). However, as most of the factors that were included within the model belonged to the domain of personality, it is therefore plausible that the obtained low loading onto cognitive ability may be an artifact ascribable to the overrepresentation of personality constructs within the model. Moreover, although most past studies investigating the relationship between cognitive ability and trait EI reported low correlations between the two constructs (O'Boyle et al., 2011), this may be due to the methods used as opposed to a true lack of a relationship. Accordingly, a study that utilized the network analysis paradigm to analyze the relationship between the two constructs indicated that they are in fact significantly connected (Zadorozhny et al., 2024).

Unlike trait EI, the latent factor loaded weakly onto ability EI (0.19). The relationship amongst trait and ability EI is commonly described as likewise modest, although much of the meta-analytic data describing the relationship between the two is significantly dated. For instance, a meta-analysis conducted in 2010 reported a correlation of *r* = 0.26 between them but predated the popularization of more recent assessments of both varieties of EI and also utilized a different categorization of emotional intelligence measures in general (Joseph and Newman, 2010). With that said, if ability EI is somewhat correlated with overall (*r* = 0.30) intelligence (Kong, 2014), while simultaneously being weakly related to trait EI (Petrides, 2011), then it is logical that the loading of the present latent factor onto it would likewise be low.

Finally, with respect to the dark traits, the latent factor loaded weakly onto narcissism (0.19). Notably, however, its loading is positive, unlike for both Machiavellianism (−0.20) and psychopathy (−0.26). A potential explanation for the positive loading onto narcissism could be its associations with traits and dispositions that are generally considered non-aversive which constitutes a pattern that is not observed with respect to the other two Dark Triad traits, such as its correlations with wellbeing and positive self-image (Giacomin and Jordan, 2016) and extraversion (O'Boyle et al., 2014). Notably, the narcissism indicator in the present study reflects only grandiose narcissism. Therefore, vulnerable narcissism was not represented in the current study.

In sum, support for shared variance amongst a range of differential psychology constructs was provided by means of a meta-meta-analytic analysis, while the uneven loadings of the latent factor onto the different constructs demonstrates that the magnitude of the correlations between the latent factor and each of the indicator variables varies considerably.

Considering the magnitude of the loading of the latent factor onto trait EI (0.96), it can be concluded that trait EI is the closest observed marker of the shared variance amongst the measures of individual differences presently examined. This thereby suggests that, within the confines of this indicator set, trait EI plays a prominent role in connecting the various aspects of differential psychology, in the sense of how they are brought into play in the pursuit and achievement of life goals and outcomes.

### 4.1 Limitations and future directions

Although the present meta-meta-analysis provides initial support for trait EI representing the shared variance amongst differential psychology constructs, there were limitations that ought to be discussed that were consequent of the exploratory nature of the present study.

It is important to note that the strong loading of trait EI (0.96) on the general factor is potentially partially ascribable to the specific methodology used in the present study, including the modeling approach and the indicators used. In particular, the final model incorporated a number of correlated residuals, which are justified theoretically and empirically but also shape the interpretation of the factor. It is also notable that the majority of the included indicators were self-report personality measures, while cognitive ability (a performance measure) was found to load only weakly onto the general factor. Therefore, future investigations are required to provide further supporting evidence for the centrality of trait EI in the domain of individual differences, as well as its link to the general factor of individual differences.

A potential limitation is that meta-analyses incorporate, by their nature, an assortment of primary studies, each of which potentially operationalizes constructs using different measures, some of which are further classified in divergent ways across different meta-analyses. In order to resolve this issue, one would need to perform meta-analyses strictly on primary studies, while incorporating the particular instrument used to operationalize a construct as a moderator. This is potentially extremely difficult to carry out in practice in cases when a great number of constructs are incorporated within one study, each of which may be measured by one of dozens of different instruments (see Supplementary Table S4).

This issue—wherein meta-analyses (and consequently, the meta-meta-analyses that rely upon them) treat potentially disparate measures as equivalent or even identical—is an enduring and fundamental concern. As Eysenck (1994) emphasized, meta-analyses can mislead by indicating that which primary studies do not show, obscuring that which they do, and generally giving rise to confusion and disorder. If, indeed, “apples and oranges” are aggregated indiscriminately, such investigations may obscure more than they illuminate. In the present study, we attempted to mitigate some of these concerns by explicitly avoiding primary study data, in order to reduce the risk of including low quality or idiosyncratic primary findings. Conversely, however, this means that we are necessarily at the mercy of the choices made by the prior meta-analysts with respect to their choices regarding construct operationalization and inclusion criteria. As such, our findings ought to be interpreted with caution, in acknowledgement of the persistent challenge of construct heterogeneity and conceptual ambiguity—not to mention the field’s fundamental flaw of relying on operationalism as a means of defining constructs in the first place (Petrides, 2019; Bringmann et al., 2022).

Most importantly, the present study was limited by its reliance on meta-analytic (as opposed to primary study) data, although this is also its strength. As noted in prior literature, populating an input correlation matrix with previously derived meta-analytic estimates along with some corresponding sample size is a potential source of bias (Oh, 2020). This is because such an approach wholly ignores effect size heterogeneity, which is likely to produce biased standard errors, thereby potentially giving rise to uninterpretable results (Jak and Cheung, 2022). In other words, conducting analyses on an input correlational matrix that consists of meta-analytic estimates (without accounting for the variance within each meta-analytic estimate) implies a loss of information that prevents one from assessing and accounting for heterogeneity.

This is a limitation that ought to be addressed in future studies, through either the extraction of primary study data for each included meta-analysis or the incorporation of true standard deviations for each effect size. Such an approach would also enable the use of modern MASEM analytical approaches including TSSEM and OSMASEM in the case of primary data and FIMASEM in the case of the latter approach (Oh, 2020; Jak et al., 2021; Cheung and Chan, 2005). As an additional benefit, this would also enable analysis on correlational matrices that contain gaps (that is, matrices that are missing correlations for certain construct pairings), which would allow for a far greater number of constructs to be incorporated in analysis (Jak and Cheung, 2017). On the other hand, the present analytical approach required a gap-free input correlational matrix which severely limited the number of constructs that could be incorporated within the analysis.

Additionally, reliance on prior meta-analyses implies accepting the limitations of the statistical approaches that were employed in prior studies. Specifically, the prevalence of basic statistical methods in extant psychology literature, especially including the utilization of simple correlations which assume linearity of relations between pairs of variables, implies a transference of that limitation to the present study. Therefore, in order to delve deeper into the true nature of relations between constructs, primary studies applying novel statistical methodologies are necessary.

It is theorized that the inclusion of additional constructs into a unifactorial model similar to the one presented herein (including constructs that were initially proposed for inclusion and for which meta-analytic data is included in Table 3) would yield evidence that would lend stronger and broader support for the theoretical latent factor proposed within this study.

Future studies may also validate the existence of the latent factor through the administration of a comprehensive questionnaire that includes measures of all described constructs to study participants (see Supplementary Table S4). Even greater validity and support could be indicated through the use of a longitudinal paradigm wherein participants would be retested using the same questionnaire at regular intervals. Additionally, the longitudinal paradigm combined with the neuroscientific approach could provide evidence that corroborates the activating role of trait EI in association with the achievement of various outcomes.

## 5 Conclusion

The present exploratory research provides initial evidence for the existence of shared variance that emerges from across the domains of differential psychology that were traditionally regarded as disparate. Due partially to the nature of traditional statistical methodology and financial incentives, the general trend in contemporary psychology has been toward the development of an ever-increasing number of measures. With that said, the present findings converge with extant correlational data but nevertheless constitute novel evidence that indicates that the various psychological aspects of individuals may not be as different from one another as is popularly considered.

This commonality may point to a quality that underscores global individual functioning, positive adjustment, and especially the ability to summon forth the aspects of individual differences that are needed for any particular situation. The finding that trait emotional intelligence and said obtained latent construct nearly entirely converge indicates that, given the present indicator set, trait EI is the best available observed proxy for the found shared variance. However, this convergence is dependent on the specific set of variables included and therefore cannot be taken as evidence of strict identity between trait EI and the purported latent factor.

The discovery of a central trait in differential psychology would correspond with ancient wisdom and the concept of eudaimonia. Per Aristotle, “if eudaimonia is activity in accordance with virtue, it is reasonable that it should be in accordance with the highest virtue; and this will be that of the best thing in us.” (Nicomachean Ethics, x.6, 1176b25).

## Data Availability

The dataset used in this study can be found at: https://osf.io/vcz46/?view_only=23c8d94fe07f4a3390bc8e8c3715f4fb.

## Footnotes

1
https://osf.io/8pfwq?view_only=f493ff7aa90145fca6897c258c6099c2
