# Supplementary material for: The shared variance amongst the measures of individual differences and trait EI: a meta-meta-analytic comparison
Source: Front Psychol. 2026 Jan 8;16:1635847. doi: 10.3389/fpsyg.2025.1635847 (PMC12823888; doi:10.3389/fpsyg.2025.1635847)
Supplement: Supplementary file 1 [file Supplementary_file_1.docx]

**Table A1**

*Loadings of Variables in Uncorrected Unifactorial Model*

| Variable | Estimate | SE of estimate | z-value | Standardized estimate |
| --- | --- | --- | --- | --- |
| Openness | 1.000 |  |  | 0.413 |
| Conscientiousness | 1.246 | .039 | 31.917 | 0.514 |
| Extraversion | 1.409 | .042 | 33.782 | 0.582 |
| Agreeableness | 1.192 | .038 | 31.218 | 0.492 |
| Neuroticism | -1.691 | .047 | -36.253 | -0.698 |
| Ability EI | 0.651 | .031 | 21.000 | 0.269 |
| Trait EI | 2.308 | .060 | 38.529 | 0.953 |
| Cognitive Ability | 0.187 | .028 | 6.747 | 0.077 |
| Machiavellianism | -0.727 | .032 | -22.828 | -0.300 |
| Narcissism | 0.447 | .029 | 15.343 | 0.185 |
| Psychopathy | -0.647 | .031 | -20.887 | -0.267 |
| Creativity | 0.845 | .033 | 25.408 | 0.349 |

*Note.* SE of Estimate represents the standard of the estimate.

**Table A2**

*Error Variances of Variables in Uncorrected Unifactorial Model*

| Variable | Estimate | SE of estimate | z-value | Standardized estimate |
| --- | --- | --- | --- | --- |
| Openness | 0.829 | .013 | 63.754 | 0.830 |
| Conscientiousness | 0.735 | .012 | 62.702 | 0.735 |
| Extraversion | 0.662 | .011 | 61.530 | 0.662 |
| Agreeableness | 0.758 | .012 | 62.988 | 0.758 |
| Neuroticism | 0.512 | .009 | 57.380 | 0.513 |
| Ability EI | 0.928 | .014 | 64.516 | 0.928 |
| Trait EI | 0.092 | .007 | 13.345 | 0.092 |
| Cognitive Ability | 0.994 | .015 | 64.904 | 0.994 |
| Machiavellianism | 0.910 | .014 | 64.397 | 0.910 |
| Narcissism | 0.966 | .015 | 64.749 | 0.966 |
| Psychopathy | 0.929 | .014 | 64.522 | 0.929 |
| Creativity | 0.878 | .014 | 64.165 | 0.878 |
| Latent Factor | 0.170 | .009 | 19.049 | 1.000 |

*Note.* SE of Estimate represents the standard of the estimate.

**Table A3**

*Loadings of Variables in the Modified Unifactorial Model*

| Variable | Estimate | SE of estimate | z-value | Standardized estimate |
| --- | --- | --- | --- | --- |
| Openness | 1.000 |  |  | 0.383 |
| Conscientiousness | 1.299 | .041 | 31.721 | 0.497 |
| Extraversion | 1.512 | .040 | 38.268 | 0.578 |
| Agreeableness | 1.205 | .038 | 32.049 | 0.461 |
| Neuroticism | -1.851 | .051 | -36.031 | -0.708 |
| Trait EI | 2.517 | .068 | 37.154 | 0.963 |
| Cognitive Ability | -0.116 | .039 | -2.970 | -0.044 |
| Ability EI | 0.496 | .033 | 15.086 | 0.190 |
| Machiavellianism | -0.513 | .034 | -14.936 | -0.196 |
| Narcissism | 0.497 | .028 | 17.854 | 0.190 |
| Psychopathy | -0.678 | .032 | -21.285 | -0.259 |
| Creativity | 0.933 | .028 | 32.856 | 0.357 |

*Note.* SE of Estimate represents the standard of the estimate.

**Table A4**

*Error Variances of Variables in the Modified Unifactorial Model*

| Variable | Estimate | SE of estimate | z-value | Standardized estimate |
| --- | --- | --- | --- | --- |
| Openness | 0.854 | .012 | 68.730 | 0.854 |
| Conscientiousness | 0.753 | .011 | 66.935 | 0.753 |
| Extraversion | 0.660 | .010 | 64.551 | 0.660 |
| Agreeableness | 0.782 | .011 | 68.887 | 0.782 |
| Neuroticism | 0.498 | .009 | 57.501 | 0.498 |
| Trait EI | 0.073 | .008 | 8.889 | 0.073 |
| Cognitive Ability | 1.002 | .015 | 68.813 | 1.002 |
| Ability EI | 0.966 | .014 | 67.761 | 0.966 |
| Machiavellianism | 0.937 | .014 | 68.473 | 0.937 |
| Narcissism | 0.941 | .013 | 74.295 | 0.941 |
| Psychopathy | 0.913 | .013 | 68.975 | 0.913 |
| Creativity | 0.854 | .013 | 67.988 | 0.854 |
| Latent Factor | 0.146 | .008 | 18.614 | 1.000 |

*Note.* SE of Estimate represents the standard of the estimate.

## **Table A5**

*Sources Utilized as Input for Meta-Meta-Analysis*

| Reference |
| --- |
| Anglim, J., Dunlop, P. D., Wee, S., Horwood, S., Wood, J. K., and Marty, A. (2022). Personality and intelligence: a meta-analysis. *Psychol. Bull.* 148, 301–336. doi: 10.1037/bul0000373  Joseph, D. L., and Newman, D. A. (2010). Emotional intelligence: an integrative meta-analysis and cascading model. *J. Appl. Psychol*. 95, 54–78. doi: 10.1037/a0017286  Karwowski, M., and Lebuda, I. (2015). The Big Five, the huge two, and creative self-beliefs: a meta-analysis. *Psychol. Aesthet. Creat. Arts* 10, 214–232.  Lebuda, I., Figura, B., and Karwowski, M. (2021). Creativity and the dark triad: a meta-analysis. *J. Res. Pers*. 92. doi: 10.1016/j.jrp.2021.104088  Michels, M. (2022). General intelligence and the dark triad. *J. Individ. Differ*. 43, 35–46. doi: 10.1027/1614-0001/a000352  Michels, M., and Schulze, R. (2021). Emotional intelligence and the dark triad: a meta-analysis. *Pers. Individ. Differ.* 180. doi: 10.1016/j.paid.2021.110961  Muris, P., Merckelbach, H., Otgaar, H., and Meijer, E. (2017). The malevolent side of human nature. *Perspect. Psychol. Sci.* 12, 183–204. doi: 10.1177/1745691616666070  O'Boyle, E. H., Forsyth, D. R., Banks, G. C., and Story, P. A. (2013). A meta-analytic review of the dark triad–intelligence connection. *J. Res. Pers*. 47, 789–794.  O'Boyle, E. H. Jr., Forsyth, D. R., Banks, G. C., Story, P. A., and White, C. D. (2014). A meta-analytic test of redundancy and relative importance of the dark triad and five-factor model of personality. *J. Pers.* 83, 644–664. doi: 10.1111/jopy.12126  O'Boyle, E. H. Jr., Humphrey, R. H., Pollack, J. M., Hawver, T. H., and Story, P. A. (2011). The relation between emotional intelligence and job performance: a meta-analysis. *J. Organ. Behav*. 32, 788–818. doi: 10.1002/job.714  Schreiber, A., and Marcus, B. (2020). The place of the "dark triad" in general models of personality: some meta-analytic clarification. *Psychol. Bull.* 146, 1021–1041. doi: 10.1037/bul0000299  Serban, A., Kepes, S., Wang, W., and Baldwin, R. (2023). Cognitive ability and creativity: typology contributions and a meta-analytic review. *Intelligence* 98. doi: 10.1016/j.intell.2023.101757  van der Linden, D., Pekaar, K. A., Bakker, A. B., Schermer, J. A., Vernon, P. A., Dunkel, C. S., et al. (2017). Overlap between the general factor of personality and emotional intelligence: a meta-analysis. *Psychol. Bull.* 143, 36–52. doi: 10.1037/bul0000078  van der Linden, D., te Nijenhuis, J., and Bakker, A. B. (2010). The general factor of personality: a meta-analysis of Big Five intercorrelations and a criterion-related validity study. *J. Res. Pers.* 44, 315–327. doi: 10.1016/j.jrp.2010.03.003  Wilmot, M. P., Wanberg, C. R., Kammeyer-Mueller, J. D., and Ones, D. S. (2019). Extraversion advantages at work: a quantitative review and synthesis of the meta-analytic evidence. *J. Appl. Psychol.* 104, 1447–1470. doi: 10.1037/apl0000415  Woo, S. E., Chernyshenko, O. S., Stark, S. E., and Conz, G. (2013). Validity of six openness facets in predicting work behaviors: a meta-analysis. *J. Pers. Assess.* 96, 76–86. doi: 10.1080/00223891.2013.806329  Xu, X., Liu, W., and Pang, W. (2019). Are emotionally intelligent people more creative? A meta-analysis of the emotional intelligence–creativity link. *Sustainability* 11. doi: 10.3390/su11216123. |
|  |
