# Supplementary material for: The shared variance amongst the measures of individual differences and trait EI: a meta-meta-analytic comparison
Source: Front Psychol. 2026 Jan 8;16:1635847. doi: 10.3389/fpsyg.2025.1635847 (PMC12823888; doi:10.3389/fpsyg.2025.1635847)
Supplement: Supplementary file 2 [file Table_1.docx]

# **Supplementary Material**

## **Table S1**

*Descriptions of Selected Articles*

| Study Reference | Brief study description |
| --- | --- |
| (Mayer et al., 2004) | Review paper on emotional intelligence and its properties, validities, and interrelations |
| (van Rooy et al., 2005) | Meta-analysis of ability and mixed (trait) EI measures and their relationships with the Big Five and cognitive intelligence |
| (Joseph and Newman, 2010) | Meta-analysis of the relationship of ability and trait EI measures with job performance, the Big Five, cognitive ability, and others |
| (Perera and DiGiacomo, 2013) | Meta-analysis assessing the relationship of trait EI and academic performance |
| (Gutiérrez-Cobo et al., 2016) | Systematic review of the relationship between ability and trait EI and cognitive tasks and academic performance |
| (van der Linden et al., 2017) | Meta-analysis establishing the link between the General Factor of Personality and trait EI |
| (Miao et al., 2019) | Meta-analysis of the relationship between trait EI and the dark triad measures |
| (Olderbak et al., 2018) | Meta-analysis of the relationship between ability EI and fluid and crystallized cognitive intelligence |
| (Stankov, 2018) | Review of the links between personality traits and cognitive ability |
| (Gong and Jiao, 2019) | Meta-analysis reviewing the trends in effect sizes between measures of ability and trait EI and other measures over time |
| (Zehetner and Zehetner, 2019) | Primary study examining purported links amongst EI, IQ, and job performance |
| (Akpur, 2020) | Systematic review assessing the relationship between academic performance and EI |
| (Alabbasi et al., 2021) | Meta-analysis of emotional intelligence amongst gifted vs. non-gifted students |
| (Do and Minbashian, 2014) | Meta-analysis examining the connection between the Big Five, GFP, and leadership outcomes |
| (MacCann et al., 2020b) | Meta-analysis of the relationship between various measures of EI and academic performance |
| (Sánchez-Álvarez et al., 2020) | Meta-analysis comparing ability and trait EI with respect to their relationship with academic performance |
| (Schilling et al., 2020) | Meta-analysis assessing the link between cognitive, ability, personality, and intentional faking in the context of candidate recruitment |
| (Cerni et al., 2021) | Primary study evaluating the impact of cognitive ability and personality on educational and life outcomes |
| (Cuadrado et al., 2021) | Meta-analysis of the impact of personality and intelligence on counterproductive academic behaviors |
| (Gignac, 2021) | Primary study assessing the link between EI and subjectively measured IQ |
| (Mammadov, 2022) | Meta-analysis of the impact of the Big Five on academic performance |
| (Ogurlu, 2021) | Meta-analysis assessing ability and trait EI performance in gifted vs. non-gifted individuals |
| (Somaa et al., 2021) | Meta-analysis examining the relationship between academic performance and EI |
| (Vashisht et al., 2021) | Systematic review and meta-analysis examining the relationships amongst personality, EI, and career adaptability in students |

## **Table S2**

*List of Target Keywords by Category*

| Intelligence | Emotional Intelligence | Personality | General |
| --- | --- | --- | --- |
| academic achievement | ability ei | agreeableness | creative achievement |
| academic performance | ability emotional intelligence | antisocial behavior | creativity |
| academic success | ability models | antisocial personality disorder | cultural intelligence |
| achievement | cool tasks | big 5 | empathy |
| aptitude | ei | big five | general ability index |
| cognition | emotion management | big five inventory | general attitude factor |
| cognitive abilities | emotion perception | big five personality traits | general cognitive ability |
| cognitive ability | emotion recognition | conscientiousness | general criteria for personality |
| cognitive assessment | emotion regulation | core self-evaluations | general factor of cognitive ability |
| cognitive competences | emotional competence | dark triad | general factor of intelligence |
| cognitive competency | emotional intelligence | emotional stability | general factor of mental ability |
| cognitive function | emotional intelligence scale | epq lie | general factor of personality |
| cognitive functioning | emotional manipulation | extraversion | general factor of psychopathology |
| cognitive intelligence | general self-efficacy | general criteria for personality | general factor of psychosocial development |
| cognitive processes | hot tasks | general factor of personality | general factor of relationship satisfaction |
| competence assessment | leadership | general personality factor | general factor of temperament |
| crystallized intelligence | mixed emotional intelligence | gfp | general intelligence factor |
| fluid ability | perceived emotional intelligence | giant 3 | general mental ability |
| fluid intelligence | self-beliefs | honesty humility | general personality factor |
| fsiq | self-efficacy | machiavellianism | general self-efficacy |
| full scale iq | self-presentation | narcissism | general social attitudes |
| g factor | social competence | neuroticism | general taste |
| g-loading | social effectiveness | openness | general values |
| general ability index | social intelligence | personality | honesty |
| general cognitive ability | teique | personality assessment | job performance |
| general factor of cognitive ability | trait ei | personality assessment inventory | job satisfaction |
| general factor of intelligence | trait emotional intelligence | personality factors | life satisfaction |
| general factor of mental ability | trait models | personality inventory | psychopathy |
| general intelligence |  | personality questionnaire | religiosity |
| general intelligence factor |  | personality testing | self-concept |
| general mental ability |  | personality tests | self-esteem |
| gifted |  | personality traits | spirituality |
| high iq |  | psychopathy | well-being |
| intellectual ability |  | psychoticism | work performance |
| intelligence |  | successful intelligence |  |
| intelligence quotient |  | temperament and character inventory |  |
| intelligence tests |  |  |  |
| iq |  |  |  |
| non-gifted |  |  |  |
| multiple intelligence |  |  |  |
|  |  |  |  |

## **Table S3**

*List of Meta-Analytical Studies* *Containing Relevant Correlations*

| Construct Pairing | Reference | N | r | rho | Data Originality |
| --- | --- | --- | --- | --- | --- |
| *Openness & Conscientiousness* | *(van der Linden et al., 2010) *** | 144117 | .14 | .20 | Original Data |
| *Openness & Extraversion* | *(van der Linden et al., 2010) *** | 144117 | .31 | .43 | Original Data |
| *Openness & Agreeableness* | *(van der Linden et al., 2010) *** | 144117 | .14 | .21 | Original Data |
| *Openness & Neuroticism* | *(van der Linden et al., 2010) *** | 144117 | -.12 | -.17 | Original Data |
| *Conscientiousness & Extraversion* | *(van der Linden et al., 2010) *** | 144117 | .21 | .29 | Original Data |
| *Conscientiousness & Agreeableness* | *(van der Linden et al., 2010) *** | 144117 | .31 | .43 | Original Data |
| *Conscientiousness & Neuroticism* | *(van der Linden et al., 2010) *** | 144117 | -.32 | -.43 | Original Data |
| *Extraversion & Agreeableness* | *(van der Linden et al., 2010) *** | 144117 | .18 | .26 | Original Data |
| *Extraversion & Neuroticism* | *(van der Linden et al., 2010) *** | 144117 | -.26 | -.36 | Original Data |
| *Neuroticism & Agreeableness* | *(van der Linden et al., 2010) *** | 144117 | -.26 | -.36 | Original Data |
| Openness & Ability EI | (Joseph and Newman, 2010) | 4155 | .18 | .21 | Original Data |
| Openness & Ability EI | (O'Boyle et al., 2011) | 4045 | .15 | .18 | Original Data |
| *Openness & Ability EI* | *(van der Linden et al., 2017) *** | 8630 | .14 | .18 | Original Data |
| Openness & Trait EI | (O'Boyle et al., 2011) | 5426 | .33 | .39 | Original Data |
| *Openness & Trait EI* | *(van der Linden et al., 2017) *** | 7626 | .31 | .38 | Original Data |
| Openness & Academic Achievement | (Connelly and Ones, 2010) | 3628 | .13 | .17 | From (Hough, 1992) |
| Openness & Academic Achievement | (Gatzka and Hell, 2018) | 50449 | .06 | .07 | Original Data |
| *Openness & Academic Achievement* | *(Mammadov, 2022) ** | 164162 | - | .10 | Original Data |
| Openness & Academic Achievement | (McAbee and Oswald, 2013) | 24996 | .07 | .08 | Original Data |
| Openness & Academic Achievement | (O'Connor and Paunonen, 2007) | 5878 | .05 | .06 | Original Data |
| Openness & Academic Achievement | (Poropat, 2009) | 28471 | - | .07 | Original Data |
| Openness & Academic Achievement | (Richardson et al., 2012) | 23096 | .09 | .09 | Original Data |
| Openness & Academic Achievement | (Salgado and Tauriz, 2012) | 6299 | .15 | .21 | Original Data |
| Openness & Academic Achievement | (Trapmann et al., 2007) | 14942 | .08 | .13 | Original Data |
| Openness & Academic Achievement | (van Aarde et al., 2017) | 1476 | -.03 | -.05 | Original Data |
| Openness & Academic Achievement | (Vedel, 2014) | 17717 | .06 | .07 | Original Data |
| Openness & Honesty-Humility | (Anglim et al., 2020) | 465 | .16 | - | Original Data |
| Openness & Honesty-Humility | (Howard and van Zandt, 2020) | 39826 | .05 | .06 | Original Data |
| Openness & Honesty-Humility | (Lee et al., 2019) | 10768 | .06 | .07 | Original Data |
| Openness & Honesty-Humility | (Schreiber and Marcus, 2020) | 40818 | .07 | - | Original Data |
| *Openness & Honesty-Humility* | *(Thielmann et al., 2021) ** | 69089 | .10 | .11 | Original Data |
| Openness & Cognitive Ability | (Anglim et al., 2022) | 83401 | - | .16 | From Supplement (Original Data) |
| Openness & Cognitive Ability | (DeNeve and Cooper, 1998) | 2546 | .05 | - | Original Data |
| Openness & Cognitive Ability | (Judge et al., 2007) | 13182 | - | .22 | Original Data |
| Openness & Cognitive Ability | (Meriac et al., 2008) | 1156 | .11 | .13 | Original Data |
| *Openness & Cognitive Ability* | *(Woo et al., 2013) *** | 85140 | .14 | .17 | Original Data |
| Openness & Cognitive Ability | (Schilling et al., 2020) | 46265 | - | .21 | Original Data |
| Openness & Machiavellianism | (Muris et al., 2017) | 8500 | -.05 | - | Original Data |
| Openness & Machiavellianism | (O'Boyle et al., 2014) | 11427 | -.04 | -.05 | Original Data |
| *Openness & Machiavellianism* | *(Schreiber and Marcus, 2020)* ******** | 40818 | -.02 | *-* | Original Data |
| Openness & Machiavellianism | (Vize et al., 2018) | 13283 | -.05 | - | Original Data |
| Openness & Narcissism | (Grijalva and Newman, 2014) | 18274 | - | .29 | From (Trzesniewski et al., 2008) |
| Openness & Narcissism | (Muris et al., 2017) | 8500 | .15 | - | Original Data |
| *Openness & Narcissism* | *(O'Boyle et al., 2014) *** | 42936 | .20 | .25 | Original Data |
| Openness & Narcissism | (Schreiber and Marcus, 2020) | 40818 | .13 | - | Original Data |
| Openness & Narcissism | (Vize et al., 2018) | 13283 | .14 | - | Original Data |
| Openness & Psychopathy | (Lilienfeld et al., 2014) | 1827 | .01 | - | Original Data |
| Openness & Psychopathy | (Muris et al., 2017) | 8500 | -.03 | - | Original Data |
| Openness & Psychopathy | (O'Boyle et al., 2014) | 23414 | .04 | .05 | Original Data |
| *Openness & Psychopathy* | *(Schreiber and Marcus, 2020) *** | 40818 | -.03 | ***-*** | Original Data |
| Openness & Psychopathy | (Vize et al., 2018) | 13283 | -.03 | - | Original Data |
| *Openness & Cultural Intelligence* | *(Schlaegel et al., 2021) ** | 1581 | .34 | .40 | Original Data |
| Openness & Job Performance | (Barrett et al., 2003) | 1434 | .03 | .05 | Original Data |
| *Openness & Job Performance* | *(Bowling, 2007) ** | 23225 | .03 | .07 | From (Barrick et al., 2001) |
| Openness & Job Performance | (Darr, 2011) | 1393 | -.01 | -.01 | Original Data |
| Openness & Job Performance | (He et al., 2019) | 4881 | .03 | .06 | Original Data |
| Openness & Job Performance | (Joseph and Newman, 2010) | 7797 | .04 | .06 | From (Hurtz and Donovan, 2000) [updated through 2008] |
| Openness & Job Performance | (Judge et al., 2007) | 7562 | - | .08 | From (Salgado, 2003) |
| Openness & Job Performance | (Mol et al., 2005) | 496 | .04 | .05 | Original Data |
| Openness & Job Performance | (Mount et al., 1998) | 1507 | .11 | .17 | Original Data |
| Openness & Job Performance | (Salgado and Tauriz, 2012) | 9519 | .07 | .11 | Original Data |
| Openness & Job Performance | (Salgado, 1997) | 2722 | .04 | .09 | Original Data |
| Openness & Job Performance | (Salgado, 1998) | 4385 | .06 | .09 | Original Data |
| Openness & Job Performance | (van Aarde et al., 2017) | 2212 | .11 | .20 | Original Data |
| Openness & Job Performance | (Vinchur et al., 1998) | 804 | .06 | .11 | Original Data |
| Openness & Job Performance | (Woo et al., 2013) | 9564 | .06 | .08 | Original Data |
| Openness & Job Performance | (Tett et al., 1991) | 1304 | .18 | .27 | Original Data |
| Openness & Job Satisfaction | (Bruk-Lee et al., 2009) | 4311 | -.02 | .02 | Original Data |
| Openness & Job Satisfaction | (Doan et al., 2021) | 3893 | .45 | .53 | Original Data |
| *Openness & Job Satisfaction* | *(Judge et al., 2002b) ** | 15196 | .01 | .02 | Original Data |
| Openness & Job Satisfaction | (Ng et al., 2005) | 10962 | - | .12 | Original Data |
| Openness & Job Satisfaction | (Steel et al., 2018) | 14621 | .03 | .04 | Original Data |
| *Openness & Creativity* | *(Karwowski and Lebuda, 2015) *** | 20823 | .47 | ***-*** | Original Data |
| Openness & Creativity | (Liu et al., 2016) | 8603 | .18 | .23 | Original Data |
| Openness & Creativity | (Ng and Feldman, 2012) | 1909 | .19 | - | Original Data |
| Openness & Creativity | (Zare and Flinchbaugh, 2018) | 9865 | .36 | .46 | Original Data |
| *Openness & Self-Efficacy* | *(Badura et al., 2020) ** | 7233 | - | .41 | Original Data |
| Openness & Self-Efficacy | (Judge et al., 2007) | 755 | - | .20 | From (Judge and Ilies, 2002) |
| Openness & Self-Efficacy | (Liu et al., 2016) | 2387 | .23 | .30 | Original Data |
| *Openness & Well-Being [Negative Affect]* | *(Anglim et al., 2020) ** | 39538 | -.05 | -.06 | Original Data |
| Openness & Well-Being [Negative Affect] | (Steel et al., 2008) | 8008 | -.02 | -.03 | Original Data |
| *Openness & Well-Being [Positive Affect]* | *(Anglim et al., 2020) ** | 41406 | .24 | .28 | Original Data |
| Openness & Well-Being [Positive Affect] | (Steel et al., 2008) | 7340 | .20 | .26 | Original Data |
| Conscientiousness & Ability EI | (Joseph and Newman, 2010) | 4155 | .12 | .13 | Original Data |
| Conscientiousness & Ability EI | (O'Boyle et al., 2011) | 4401 | .10 | .11 | Original Data |
| *Conscientiousness & Ability EI* | *(van der Linden et al., 2017)* ******** | 8630 | .09 | .11 | Original Data |
| Conscientiousness & Trait EI | (O'Boyle et al., 2011) | 6149 | .32 | .38 | Original Data |
| *Conscientiousness & Trait EI* | *(van der Linden et al., 2017)* ******** | *7626* | *.40* | *.47* | Original Data |
| Conscientiousness & Academic Achievement | (Connelly and Ones, 2010) | 18661 | .23 | .31 | From (Hough, 1992) |
| *Conscientiousness & Academic Achievement* | *(Mammadov, 2022) ** | 169411 | - | .26 | Original Data |
| Conscientiousness & Academic Achievement | (McAbee and Oswald, 2013) | 26382 | .22 | .26 | Original Data |
| Conscientiousness & Academic Achievement | (O'Connor and Paunonen, 2007) | 5878 | .22 | .24 | Original Data |
| Conscientiousness & Academic Achievement | (Poropat, 2009) | 32887 | - | .23 | Original Data |
| Conscientiousness & Academic Achievement | (Richardson et al., 2012) | 27875 | .19 | .23 | Original Data |
| Conscientiousness & Academic Achievement | (Salgado and Tauriz, 2012) | 6314 | .12 | .19 | Original Data |
| Conscientiousness & Academic Achievement | (Trapmann et al., 2007) | 10855 | .22 | .27 | Original Data |
| Conscientiousness & Academic Achievement | (van Aarde et al., 2017) | 1464 | .18 | .28 | Original Data |
| Conscientiousness & Academic Achievement | (Vedel, 2014) | 17717 | .28 | .26 | Original Data |
| Conscientiousness & Honesty-Humility | (Anglim et al., 2020) | 465 | .32 | - | Original Data |
| Conscientiousness & Honesty-Humility | (Howard and van Zandt, 2020) | 42857 | .20 | .25 | Original Data |
| Conscientiousness & Honesty-Humility | (Lee et al., 2019) | 14445 | .20 | .23 | Original Data |
| Conscientiousness & Honesty-Humility | (Schreiber and Marcus, 2020) | 40818 | .14 | - | Original Data |
| *Conscientiousness & Honesty-Humility* | *(Thielmann et al., 2021)* | 68546 | .20 | .24 | Original Data |
| *Conscientiousness & Cognitive Ability* | *(Anglim et al., 2022) *** | 86091 | - | -.03 | Original Data |
| Conscientiousness & Cognitive Ability | (Cortina et al., 2000) | 53144 | .05 | .08 | Original Data |
| Conscientiousness & Cognitive Ability | (Judge et al., 2007) | 15429 | - | -.04 | Original Data |
| Conscientiousness & Cognitive Ability | (Meriac et al., 2008) | 690 | .20 | .24 | Original Data |
| Conscientiousness & Cognitive Ability | (Brown et al., 2011) | 4850 | - | .02 | From (Ackerman and Heggestad, 1997) |
| Conscientiousness & Cognitive Ability | (Schilling et al., 2020) | 46265 | - | -.02 | Original Data |
| Conscientiousness & Machiavellianism | (Muris et al., 2017) | 8500 | -.25 | - | Original Data |
| Conscientiousness & Machiavellianism | (O'Boyle et al., 2014) | 12131 | -.21 | -.27 | Original Data |
| *Conscientiousness & Machiavellianism* | *(Schreiber and Marcus, 2020) *** | 40818 | -.19 | ***-*** | Original Data |
| Conscientiousness & Machiavellianism | (Vize et al., 2018) | 13283 | -.21 | - | Original Data |
| Conscientiousness & Narcissism | (Grijalva and Newman, 2014) | 18274 | - | .14 | From (Trzesniewski et al., 2008) |
| Conscientiousness & Narcissism | (Muris et al., 2017) | 8500 | -.01 | - | Original Data |
| *Conscientiousness & Narcissism* | *(O'Boyle et al., 2014) *** | 43707 | .09 | .11 | Original Data |
| Conscientiousness & Narcissism | (Schreiber and Marcus, 2020) | 40818 | .00 | - | Original Data |
| Conscientiousness & Narcissism | (Vize et al., 2018) | 13283 | -.01 | - | Original Data |
| Conscientiousness & Psychopathy | (Lilienfeld et al., 2014) | 1827 | -.14 | - | Original Data |
| Conscientiousness & Psychopathy | (Muris et al., 2017) | 8500 | -.27 | - | Original Data |
| Conscientiousness & Psychopathy | (O'Boyle et al., 2014) | 23528 | -.31 | -.39 | Original Data |
| Conscientiousness & *Psychopathy* | *(Schreiber and Marcus, 2020) *** | 40818 | -.24 | ***-*** | Original Data |
| Conscientiousness & Psychopathy | (Vize et al., 2018) | 13283 | -.26 | - | Original Data |
| *Conscientiousness & Cultural Intelligence* | *(Schlaegel et al., 2021) ** | 853 | .20 | .17 | Original Data |
| Conscientiousness & Job Performance | (Barrett et al., 2003) | 1731 | .04 | .06 | Original Data |
| *Conscientiousness & Job Performance* | *(Bowling, 2007) ** | 48100 | .12 | .27 | From (Barrick et al., 2001) |
| Conscientiousness & Job Performance | (Brown et al., 2011) | 12893 | - | .21 | From (Barrick and Mount, 1991) |
| Conscientiousness & Job Performance | (Darr, 2011) | 1774 | .23 | .35 | Original Data |
| Conscientiousness & Job Performance | (Doan et al., 2021) | 1374 | .23 | .29 | Original Data |
| Conscientiousness & Job Performance | (He et al., 2019) | 7342 | .15 | .24 | Original Data |
| Conscientiousness & Job Performance | (Joseph and Newman, 2010) | 12434 | .15 | .21 | From (Hurtz and Donovan, 2000) [updated through 2008] |
| Conscientiousness & Job Performance | (Judge et al., 2007) | 19460 | - | .28 | From (Salgado, 2003) |
| Conscientiousness & Job Performance | (Meriac et al., 2008) | 128 | .24 | .29 | Original Data |
| Conscientiousness & Job Performance | (Mol et al., 2005) | 496 | .12 | .14 | Original Data |
| Conscientiousness & Job Performance | (Mount et al., 1998) | 1586 | .17 | .26 | Original Data |
| Conscientiousness & Job Performance | (Salgado and Tauriz, 2012) | 13122 | .11 | .20 | Original Data |
| Conscientiousness & Job Performance | (Salgado, 1997) | 3295 | .10 | .25 | Original Data |
| Conscientiousness & Job Performance | (Salgado, 1998) | 4985 | .13 | .23 | Original Data |
| Conscientiousness & Job Performance | (van Aarde et al., 2017) | 2313 | .04 | .08 | Original Data |
| Conscientiousness & Job Performance | (Vinchur et al., 1998) | 2186 | .11 | .21 | Original Data |
| Conscientiousness & Job Performance | (Watrin et al., 2022) | 23305 | .17 | - | Original Data |
| Conscientiousness & Job Performance | (Kepes and McDaniel, 2015) | 19625 | .16 | - | Original Data |
| Conscientiousness & Job Performance | (Tett et al., 1991) | 450 | .12 | .18 | Original Data |
| Conscientiousness &  Job Satisfaction | (Bruk-Lee et al., 2009) | 7630 | .16 | .07 | Original Data |
| Conscientiousness &  Job Satisfaction | (Doan et al., 2021) | 744 | .16 | .22 | Original Data |
| *Conscientiousness &*  *Job Satisfaction* | *(Judge et al., 2002b) ** | 21719 | .20 | .26 | Original Data |
| Conscientiousness &  Job Satisfaction | (Ng et al., 2005) | 10566 | - | .14 | Original Data |
| Conscientiousness &  Job Satisfaction | (Steel et al., 2018) | 14953 | .16 | .20 | Original Data |
| *Conscientiousness & Creativity* | *(Karwowski and Lebuda, 2015) *** | 20300 | .13 | *-* | Original Data |
| Conscientiousness & Creativity | (Liu et al., 2016) | 4299 | .10 | .13 | Original Data |
| Conscientiousness & Creativity | (Zare and Flinchbaugh, 2018) | 8803 | .14 | .18 | Original Data |
| Conscientiousness & Self-Efficacy | (Badura et al., 2020) | 8322 | - | .34 | Original Data |
| *Conscientiousness & Self-Efficacy* | *(Joseph et al., 2015b) ** | 10027 | .45 | .54 | Original Data |
| Conscientiousness & Self-Efficacy | (Judge et al., 2007) | 3483 | - | .22 | From (Judge and Ilies, 2002) |
| Conscientiousness & Self-Efficacy | (Liu et al., 2016) | 1201 | .30 | .38 | Original Data |
| *Conscientiousness & Well-Being [Negative Affect]* | *(Anglim et al., 2020) ** | 42358 | -.25 | -.29 | Original Data |
| Conscientiousness & Well-Being [Negative Affect] | (Steel et al., 2008) | 7749 | -.20 | -.26 | Original Data |
| *Conscientiousness & Well-Being [Positive Affect]* | *(Anglim et al., 2020) ** | 43497 | .35 | .40 | Original Data |
| Conscientiousness & Well-Being [Positive Affect] | (Steel et al., 2008) | 5976 | .27 | .31 | Original Data |
| Extraversion & Ability EI | (Joseph and Newman, 2010) | 4269 | .15 | .18 | Original Data |
| Extraversion & Ability EI | (O'Boyle et al., 2011) | 4684 | .09 | .11 | Original Data |
| *Extraversion & Ability EI* | *(van der Linden et al., 2017) *** | 8630 | .05 | .06 | Original Data |
| Extraversion & Trait EI | (O'Boyle et al., 2011) | 6655 | .42 | .49 | Original Data |
| *Extraversion & Trait EI* | *(van der Linden et al., 2017) *** | 7626 | .47 | .56 | Original Data |
| Extraversion & Academic Achievement | (Connelly and Ones, 2010) | 63057 | .07 | .09 | Original Data |
| *Extraversion & Academic Achievement* | *(Mammadov, 2022) ** | 162663 | - | -.01 | Original Data |
| Extraversion & Academic Achievement | (McAbee and Oswald, 2013) | 24740 | -.02 | -.03 | Original Data |
| Extraversion & Academic Achievement | (O'Connor and Paunonen, 2007) | 5161 | -.05 | -.05 | Original Data |
| Extraversion & Academic Achievement | (Poropat, 2009) | 28424 | - | -.01 | Original Data |
| Extraversion & Academic Achievement | (Richardson et al., 2012) | 23730 | -.04 | -.03 | Original Data |
| Extraversion & Academic Achievement | (Salgado and Tauriz, 2012) | 6884 | -.02 | -.03 | Original Data |
| Extraversion & Academic Achievement | (Trapmann et al., 2007) | 12424 | .01 | -.06 | Original Data |
| Extraversion & Academic Achievement | (van Aarde et al., 2017) | 1480 | -.26 | -.38 | Original Data |
| Extraversion & Academic Achievement | (Vedel, 2014) | 17717 | .00 | .00 | Original Data |
| Extraversion & Academic Achievement | (Wilmot et al., 2019) | 32969 | -.04 | -.05 | Original Data |
| Extraversion & Honesty-Humility | (Anglim et al., 2020) | 465 | -.09 | - | Original Data |
| Extraversion & Honesty-Humility | (Howard and van Zandt, 2020) | 39977 | -.06 | -.08 | Original Data |
| Extraversion & Honesty-Humility | (Lee et al., 2019) | 12636 | -.07 | -.09 | Original Data |
| Extraversion & Honesty-Humility | (Schreiber and Marcus, 2020) | 40818 | .02 | - | Original Data |
| *Extraversion & Honesty-Humility* | *(Thielmann et al., 2021) ** | 68857 | -.04 | -.05 | Original Data |
| *Extraversion & Cognitive Ability* | *(Anglim et al., 2022) *** | 82023 | - | -.02 | Original Data |
| Extraversion & Cognitive Ability | (Judge et al., 2007) | 21602 | - | .02 | Original Data |
| Extraversion & Cognitive Ability | (Meriac et al., 2008) | 1166 | .06 | .08 | Original Data |
| Extraversion & Cognitive Ability | (Wolf and Ackerman, 2005) | 23053 | - | .05 | Original Data |
| Extraversion & Cognitive Ability | (Schilling et al., 2020) | 46265 | - | .00 | Original Data |
| Extraversion & Machiavellianism | (Muris et al., 2017) | 8500 | -.08 | - | Original Data |
| Extraversion & Machiavellianism | (O'Boyle et al., 2014) | 13187 | -.01 | -.01 | Original Data |
| *Extraversion & Machiavellianism* | *(Schreiber and Marcus, 2020) *** | 40818 | -.02 | - | Original Data |
| Extraversion & Machiavellianism | (Vize et al., 2018) | 13587 | -.04 | - | Original Data |
| Extraversion & Narcissism | (Grijalva and Newman, 2014) | 18274 | - | .59 | From (Trzesniewski et al., 2008) |
| Extraversion & Narcissism | (Grijalva et al., 2015) | 28345 | .45 | .55 | Original Data |
| Extraversion & Narcissism | (Muris et al., 2017) | 8500 | .31 | - | Original Data |
| *Extraversion & Narcissism* | *(O'Boyle et al., 2014) *** | 44237 | .40 | .49 | Original Data |
| Extraversion & Narcissism | (Schreiber and Marcus, 2020) | 40818 | .31 | - | Original Data |
| Extraversion & Narcissism | (Vize et al., 2018) | 13587 | .32 | - | Original Data |
| Extraversion & Psychopathy | (Lilienfeld et al., 2014) | 1827 | .02 | - | Original Data |
| Extraversion & Psychopathy | (Muris et al., 2017) | 8500 | .01 | - | Original Data |
| Extraversion & Psychopathy | (O'Boyle et al., 2014) | 25060 | .04 | .05 | Original Data |
| *Extraversion & Psychopathy* | *(Schreiber and Marcus, 2020) *** | 40818 | .01 | ***-*** | Original Data |
| Extraversion & Psychopathy | (Vize et al., 2018) | 13587 | .02 | - | Original Data |
| *Extraversion & Cultural Intelligence* | *(Schlaegel et al., 2021) ** | 977 | .28 | .32 | Original Data |
| Extraversion & Job Performance | (Barrett et al., 2003) | 1231 | .04 | .04 | Original Data |
| Extraversion & Job Performance | (Bowling, 2007) | 39432 | .06 | .15 | From (Barrick et al., 2001) |
| Extraversion & Job Performance | (Darr, 2011) | 1774 | .13 | .19 | Original Data |
| Extraversion & Job Performance | (Doan et al., 2021) | 1133 | .19 | .23 | Original Data |
| Extraversion & Job Performance | (He et al., 2019) | 5809 | .06 | .09 | Original Data |
| Extraversion & Job Performance | (Joseph and Newman, 2010) | 9664 | .06 | .09 | From (Hurtz and Donovan, 2000) [updated through 2008] |
| Extraversion & Job Performance | (Judge et al., 2007) | 11940 | - | .12 | From (Salgado, 2003) |
| Extraversion & Job Performance | (Meriac et al., 2008) | 174 | .24 | .29 | Original Data |
| Extraversion & Job Performance | (Mol et al., 2005) | 586 | .15 | .20 | Original Data |
| Extraversion & Job Performance | (Mount et al., 1998) | 1507 | .09 | .14 | Original Data |
| Extraversion & Job Performance | (Salgado and Tauriz, 2012) | 11445 | .07 | .12 | Original Data |
| Extraversion & Job Performance | (Salgado, 1997) | 3806 | .05 | .12 | Original Data |
| Extraversion & Job Performance | (Salgado, 1998) | 6098 | .10 | .15 | Original Data |
| Extraversion & Job Performance | (van Aarde et al., 2017) | 2523 | .09 | .16 | Original Data |
| Extraversion & Job Performance | (Vinchur et al., 1998) | 3112 | .09 | .18 | Original Data |
| *Extraversion & Job Performance* | *(Wilmot et al., 2019) ** | 71016 | .12 | .18 | Original Data |
| Extraversion & Job Performance | (Tett et al., 1991) | 2302 | .10 | .16 | Original Data |
| Extraversion & Job Satisfaction | (Bowling et al., 2008) | 1923 | .09 | .11 | Original Data |
| Extraversion & Job Satisfaction | (Bruk-Lee et al., 2009) | 9003 | .12 | .14 | Original Data |
| Extraversion & Job Satisfaction | (Doan et al., 2021) | 994 | .29 | .40 | Original Data |
| Extraversion & Job Satisfaction | (Judge et al., 2002b) | 20184 | .19 | .25 | Original Data |
| Extraversion & Job Satisfaction | (Ng et al., 2005) | 10566 | - | .27 | Original Data |
| Extraversion & Job Satisfaction | (Steel et al., 2018) | 13805 | .18 | .23 | Original Data |
| *Extraversion & Job Satisfaction* | *(Wilmot et al., 2019) ** | 21802 | .19 | .22 | Original Data |
| Extraversion & Creativity | (Karwowski and Lebuda, 2015) | 19861 | .26 | - | Original Data |
| *Extraversion & Creativity* | *(Wilmot et al., 2019) *** | 38600 | .13 | .18 | Original Data |
| Extraversion & Creativity | (Zare and Flinchbaugh, 2018) | 8357 | .21 | .27 | Original Data |
| Extraversion & Self-Efficacy | (Badura et al., 2020) | 8475 | - | .46 | Original Data |
| *Extraversion & Self-Efficacy* | *(Joseph et al., 2015b) ** | 8479 | .42 | .51 | Original Data |
| Extraversion & Self-Efficacy | (Judge et al., 2007) | 755 | - | .33 | From (Judge and Ilies, 2002) |
| Extraversion & Self-Efficacy | (Wilmot et al., 2019) | 2067 | .24 | .31 | Original Data |
| *Extraversion & Well-Being [Negative Affect]* | *(Anglim et al., 2020) ** | 49212 | -.21 | -.24 | Original Data |
| Extraversion & Well-Being [Negative Affect] | (Joseph et al., 2015a) | 4457 | - | -.30 | From (Watson et al., 1999) |
| Extraversion & Well-Being [Negative Affect] | (Steel et al., 2008) | 11569 | -.18 | -.23 | Original Data |
| *Extraversion & Well-Being [Positive Affect]* | *(Anglim et al., 2020) ** | 51731 | .44 | .51 | Original Data |
| Extraversion & Well-Being [Positive Affect] | (DeNeve and Cooper, 1998) | 1117 | .31 | - | Original Data |
| Extraversion & Well-Being [Positive Affect] | (Joseph et al., 2015a) | 4457 | - | .62 | From (Watson et al., 1999) |
| Extraversion & Well-Being [Positive Affect] | (Steel et al., 2008) | 12898 | .44 | .54 | Original Data |
| Agreeableness & Ability EI | (Joseph and Newman, 2010) | 4287 | .25 | .29 | Original Data |
| Agreeableness & Ability EI | (O'Boyle et al., 2011) | 3998 | .22 | .26 | Original Data |
| *Agreeableness & Ability EI* | *(van der Linden et al., 2017) *** | 8630 | .16 | .20 | Original Data |
| Agreeableness &  Trait EI | (O'Boyle et al., 2011) | 5992 | .32 | .38 | Original Data |
| *Agreeableness &*  *Trait EI* | *(van der Linden et al., 2017) *** | 7626 | .37 | .45 | Original Data |
| Agreeableness & Academic Achievement | (Connelly and Ones, 2010) | 7330 | .01 | .01 | From (Hough, 1992) |
| *Agreeableness & Academic Achievement* | *(Mammadov, 2022) ** | 163029 | - | .08 | Original Data |
| Agreeableness & Academic Achievement | (McAbee and Oswald, 2013) | 24615 | .06 | .07 | Original Data |
| Agreeableness & Academic Achievement | (O'Connor and Paunonen, 2007) | 4490 | .06 | .06 | Original Data |
| Agreeableness & Academic Achievement | (Poropat, 2009) | 27944 | - | .06 | Original Data |
| Agreeableness & Academic Achievement | (Richardson et al., 2012) | 21734 | .07 | .06 | Original Data |
| Agreeableness & Academic Achievement | (Salgado and Tauriz, 2012) | 6560 | -.06 | -.09 | Original Data |
| Agreeableness & Academic Achievement | (Trapmann et al., 2007) | 12452 | .04 | .06 | Original Data |
| Agreeableness & Academic Achievement | (van Aarde et al., 2017) | 1478 | -.04 | -.06 | Original Data |
| Agreeableness & Academic Achievement | (Vedel, 2014) | 17717 | .09 | .08 | Original Data |
| Agreeableness & Honesty-Humility | (Anglim et al., 2020) | 465 | .67 | - | Original Data |
| Agreeableness & Honesty-Humility | (Howard and van Zandt, 2020) | 44267 | .40 | .50 | Original Data |
| Agreeableness & Honesty-Humility | (Lee et al., 2019) | 15692 | .43 | .54 | Original Data |
| Agreeableness & Honesty-Humility | (Schreiber and Marcus, 2020) | 40818 | .40 | - | Original Data |
| *Agreeableness & Honesty-Humility* | *(Thielmann et al., 2021) ** | 69905 | .40 | .47 | Original Data |
| *Agreeableness & Cognitive Ability* | *(Anglim et al., 2022) *** | 81621 | - | .00 | Original Data |
| Agreeableness & Cognitive Ability | (Judge et al., 2007) | 11190 | - | .00 | Original Data |
| Agreeableness & Cognitive Ability | (Meriac et al., 2008) | 1227 | .12 | .15 | Original Data |
| Agreeableness & Cognitive Ability | (Schilling et al., 2020) | 46265 | - | .01 | Original Data |
| Agreeableness & Machiavellianism | (Muris et al., 2017) | 8500 | -.43 | - | Original Data |
| Agreeableness & Machiavellianism | (O'Boyle et al., 2014) | 11326 | -.39 | -.50 | Original Data |
| *Agreeableness & Machiavellianism* | *(Schreiber and Marcus, 2020) *** | 40818 | -.40 | ***-*** | Original Data |
| Agreeableness & Machiavellianism | (Vize et al., 2018) | 14011 | -.43 | - | Original Data |
| Agreeableness & Narcissism | (Grijalva and Newman, 2014) | 18274 | - | -.18 | From (Trzesniewski et al., 2008) |
| Agreeableness & Narcissism | (Muris et al., 2017) | 8500 | -.21 | - | Original Data |
| *Agreeableness & Narcissism* | *(O'Boyle et al., 2014) *** | 44480 | -.29 | -.36 | Original Data |
| Agreeableness & Narcissism | (Schreiber and Marcus, 2020) | 40818 | -.18 | - | Original Data |
| Agreeableness & Narcissism | (Vize et al., 2018) | 14011 | -.22 | - | Original Data |
| Agreeableness & Psychopathy | (Lilienfeld et al., 2014) | 1827 | -.32 | - | Original Data |
| Agreeableness & Psychopathy | (Muris et al., 2017) | 8500 | -.46 | - | Original Data |
| Agreeableness & Psychopathy | (O'Boyle et al., 2014) | 23216 | -.42 | -.53 | Original Data |
| *Agreeableness & Psychopathy* | *(Schreiber and Marcus, 2020) *** | 40818 | -.47 | ***-*** | Original Data |
| Agreeableness & Psychopathy | (Vize et al., 2018) | 14011 | -.45 | - | Original Data |
| *Agreeableness & Cultural Intelligence* | *(Schlaegel et al., 2021) ** | 853 | .12 | .14 | Original Data |
| Agreeableness & Job Performance | (Barrett et al., 2003) | 1231 | .02 | .03 | Original Data |
| *Agreeableness & Job Performance* | *(Bowling, 2007) ** | 36210 | .06 | .13 | From (Barrick et al., 2001) |
| Agreeableness & Job Performance | (Darr, 2011) | 1393 | .09 | .13 | Original Data |
| Agreeableness & Job Performance | (Doan et al., 2021) | 1133 | .18 | .23 | Original Data |
| Agreeableness & Job Performance | (He et al., 2019) | 5803 | .07 | .12 | Original Data |
| Agreeableness & Job Performance | (Joseph and Newman, 2010) | 9702 | .05 | .07 | From (Hurtz and Donovan, 2000) [updated through 2008] |
| Agreeableness & Job Performance | (Judge et al., 2007) | 10716 | - | .13 | From (Salgado, 2003) |
| Agreeableness & Job Performance | (Mol et al., 2005) | 494 | .18 | .23 | Original Data |
| Agreeableness & Job Performance | (Mount et al., 1998) | 1586 | .14 | .21 | Original Data |
| Agreeableness & Job Performance | (Salgado and Tauriz, 2012) | 10611 | .03 | .05 | Original Data |
| Agreeableness & Job Performance | (Salgado, 1997) | 3466 | .01 | .02 | Original Data |
| Agreeableness & Job Performance | (Salgado, 1998) | 5174 | .01 | .02 | Original Data |
| Agreeableness & Job Performance | (van Aarde et al., 2017) | 2212 | .00 | .00 | Original Data |
| Agreeableness & Job Performance | (Vinchur et al., 1998) | 2342 | .03 | .06 | Original Data |
| Agreeableness & Job Performance | (Tett et al., 1991) | 280 | .22 | .33 | Original Data |
| Agreeableness & Job Satisfaction | (Bruk-Lee et al., 2009) | 4800 | .13 | .09 | Original Data |
| Agreeableness & Job Satisfaction | (Doan et al., 2021) | 744 | .11 | .14 | Original Data |
| Agreeableness & Job Satisfaction | (Judge et al., 2002b) | 11856 | .13 | .17 | Original Data |
| Agreeableness & Job Satisfaction | (Ng et al., 2005) | 4634 | - | .11 | Original Data |
| *Agreeableness & Job Satisfaction* | *(Steel et al., 2018) ** | 12181 | .12 | .16 | Original Data |
| *Agreeableness & Creativity* | *(Karwowski and Lebuda, 2015) *** | 19861 | .07 | *-* | Original Data |
| Agreeableness & Creativity | (Zare and Flinchbaugh, 2018) | 7846 | .06 | .08 | Original Data |
| *Agreeableness & Self-Efficacy* | *(Badura et al., 2020) ** | 7269 | - | .27 | Original Data |
| Agreeableness & Self-Efficacy | (Judge et al., 2007) | 1099 | - | .11 | From (Judge and Ilies, 2002) |
| *Agreeableness & Well-Being [Negative Affect]* | *(Anglim et al., 2020) ** | 39023 | -.25 | -.30 | Original Data |
| Agreeableness & Well-Being [Negative Affect] | (Steel et al., 2008) | 7306 | -.20 | -.26 | Original Data |
| *Agreeableness & Well-Being [Positive Affect]* | *(Anglim et al., 2020) ** | 40714 | .19 | .22 | Original Data |
| Agreeableness & Well-Being [Positive Affect] | (Steel et al., 2008) | 6040 | .12 | .15 | Original Data |
| Neuroticism &  Ability EI | (Joseph and Newman, 2010) | 4401 | -.17 | -.20 | Original Data |
| Neuroticism &  Ability EI | (O'Boyle et al., 2011) | 4596 | -.13 | -.16 | Original Data |
| *Neuroticism &*  *Ability EI* | *van der Linden et al. (2017) *** | 8630 | -.09 | -.11 | Original Data |
| Neuroticism &  Trait EI | (O'Boyle et al., 2011) | 6829 | -.47 | -.54 | Original Data |
| *Neuroticism &*  *Trait EI* | *van der Linden et al. (2017) *** | 7626 | -.58 | -.68 | Original Data |
| Neuroticism & Academic Achievement | (Connelly and Ones, 2010) | 70588 | -.20 | -.25 | From (Hough, 1992) |
| *Neuroticism & Academic Achievement* | *(Mammadov, 2022) ** | 164092 | - | -.02 | Original Data |
| Neuroticism & Academic Achievement | (McAbee and Oswald, 2013) | 24968 | .00 | .00 | Original Data |
| Neuroticism & Academic Achievement | (O'Connor and Paunonen, 2007) | 5091 | -.03 | -.03 | Original Data |
| Neuroticism & Academic Achievement | (Poropat, 2009) | 28967 | - | -.01 | Original Data |
| Neuroticism & Academic Achievement | (Richardson et al., 2012) | 23659 | -.01 | .01 | Original Data |
| Neuroticism & Academic Achievement | (Salgado and Tauriz, 2012) | 3916 | -.03 | -.05 | Original Data |
| Neuroticism & Academic Achievement | (Trapmann et al., 2007) | 14653 | -.04 | .00 | Original Data |
| Neuroticism & Academic Achievement | (van Aarde et al., 2017) | 1478 | .02 | .03 | Original Data |
| Neuroticism & Academic Achievement | (Vedel, 2014) | 17717 | -.02 | -.01 | Original Data |
| Neuroticism & Honesty-Humility | (Anglim et al., 2020) | 465 | -.19 | - | Original Data |
| Neuroticism & Honesty-Humility | (Howard and van Zandt, 2020) | 42095 | -.11 | -.14 | Original Data |
| Neuroticism & Honesty-Humility | (Lee et al., 2019) | 14341 | -.11 | -.13 | Original Data |
| Neuroticism & Honesty-Humility | (Schreiber and Marcus, 2020) | 40818 | -.07 | - | Original Data |
| *Neuroticism & Honesty-Humility* | *(Thielmann et al., 2021) ** | 69372 | -.11 | -.13 | Original Data |
| *Neuroticism &* Psychoticism | *(DeNeve and Cooper, 1998) ** | 419 | -.06 | - | Original Data |
| *Neuroticism & Cognitive Ability* | *(Anglim et al., 2022) *** | 85357 | - | -.07 | Original Data |
| Neuroticism & Cognitive Ability | (Judge et al., 2007) | 21404 | - | -.09 | Original Data |
| Neuroticism & Cognitive Ability | (Meriac et al., 2008) | 725 | -.07 | -.08 | Original Data |
| Neuroticism & Cognitive Ability | (Schilling et al., 2020) | 46265 | - | -.06 | Original Data |
| Neuroticism & Machiavellianism | (Muris et al., 2017) | 8500 | .07 | - | Original Data |
| Neuroticism & Machiavellianism | (O'Boyle et al., 2014) | 13977 | .09 | .11 | Original Data |
| *Neuroticism & Machiavellianism* | *(Schreiber and Marcus, 2020) *** | 40818 | .13 | - | Original Data |
| Neuroticism & Machiavellianism | (Vize et al., 2018) | 14135 | .06 | - | Original Data |
| Neuroticism & Narcissism | (Grijalva and Newman, 2014) | 18274 | - | -.24 | From (Trzesniewski et al., 2008) |
| Neuroticism & Narcissism | (Muris et al., 2017) | 8500 | -.04 | - | Original Data |
| *Neuroticism & Narcissism* | *(O'Boyle et al., 2014) *** | 45885 | -.16 | -.20 | Original Data |
| Neuroticism & Narcissism | (Schreiber and Marcus, 2020) | 40818 | -.05 | - | Original Data |
| Neuroticism & Narcissism | (Vize et al., 2018) | 14135 | .00 | - | Original Data |
| Neuroticism & Psychopathy | (Lilienfeld et al., 2014) | 1827 | .06 | - | Original Data |
| Neuroticism & Psychopathy | (Muris et al., 2017) | 8500 | -.07 | - | Original Data |
| Neuroticism & Psychopathy | (O'Boyle et al., 2014) | 25465 | .05 | .06 | Original Data |
| *Neuroticism & Psychopathy* | *(Schreiber and Marcus, 2020) *** | 40818 | .08 | *-* | Original Data |
| Neuroticism & Psychopathy | (Vize et al., 2018) | 14135 | .02 | - | Original Data |
| Neuroticism &  *Cultural Intelligence* | *(Schlaegel et al., 2021) ** | 853 | -.06 | -.06 | Original Data |
| Neuroticism &  Job Performance | (Barrett et al., 2003) | 1231 | -.07 | -.08 | Original Data |
| *Neuroticism & Job Performance* | *(Bowling, 2007) ** | 38817 | -.06 | -.13 | From (Barrick et al., 2001) |
| Neuroticism & Job Performance | (Darr, 2011) | 1774 | -.16 | -.22 | Original Data |
| Neuroticism & Job Performance | (Doan et al., 2021) | 852 | -.21 | -.26 | Original Data |
| Neuroticism & Job Performance | (He et al., 2019) | 5027 | -.09 | -.15 | Original Data |
| Neuroticism & Job Performance | (Joseph and Newman, 2010) | 9184 | -.08 | -.11 | From (Hurtz and Donovan, 2000) [updated through 2008] |
| Neuroticism & Job Performance | (Judge and Bono, 2001) | 4106 | -.16 | -.19 | Original Data |
| Neuroticism & Job Performance | (Judge et al., 2007) | 10786 | - | -.16 | From (Salgado, 2003) |
| Neuroticism & Job Performance | (Mol et al., 2005) | 497 | -.05 | -.06 | Original Data |
| Neuroticism & Job Performance | (Mount et al., 1998) | 1586 | -.12 | -.18 | Original Data |
| Neuroticism & Job Performance | (Salgado and Tauriz, 2012) | 9775 | -.03 | -.06 | Original Data |
| Neuroticism & Job Performance | (Salgado, 1997) | 3877 | -.09 | -.19 | Original Data |
| Neuroticism & Job Performance | (Salgado, 1998) | 6383 | -.15 | -.23 | Original Data |
| Neuroticism & Job Performance | (van Aarde et al., 2017) | 2317 | -.13 | -.21 | Original Data |
| Neuroticism & Job Performance | (Vinchur et al., 1998) | 3134 | -.05 | -.10 | Original Data |
| Neuroticism & Job Performance | (Tett et al., 1991) | 900 | -.15 | -.22 | Original Data |
| Neuroticism & Job Satisfaction | (Bowling et al., 2008) | 2044 | -.21 | -.25 | Original Data |
| Neuroticism &  Job Satisfaction | (Bruk-Lee et al., 2009) | 9183 | -.25 | -.25 | Original Data |
| Neuroticism &  Job Satisfaction | (Doan et al., 2021) | 1166 | -.05 | -.05 | Original Data |
| Neuroticism &  Job Satisfaction | (Judge and Bono, 2001) | 7658 | -.20 | -.24 | Original Data |
| *Neuroticism &*  *Job Satisfaction* | *(Judge et al., 2002b) ** | 24527 | -.24 | -.29 | Original Data |
| Neuroticism &  Job Satisfaction | (Ng et al., 2005) | 10566 | - | -.36 | Original Data |
| Neuroticism &  Job Satisfaction | (Steel et al., 2018) | 17347 | -.21 | -.26 | Original Data |
| *Neuroticism &*  *Creativity* | *(Karwowski and Lebuda, 2015) *** | 19861 | -.12 | - | Original Data |
| Neuroticism &  Creativity | (Zare and Flinchbaugh, 2018) | 7661 | -.07 | -.08 | Original Data |
| Neuroticism & Self-Efficacy | (Badura et al., 2020) | 7973 | - | -.32 | Original Data |
| Neuroticism & Self-Efficacy | (Bowling, 2007) | 1541 | - | -.62 | From (Judge et al., 2002a) |
| *Neuroticism & Self-Efficacy* | *(Joseph et al., 2015b) ** | 12510 | -.48 | -.56 | Original Data |
| Neuroticism & Self-Efficacy | (Judge et al., 2007) | 6730 | - | -.35 | From (Judge and Ilies, 2002) |
| *Neuroticism* & *Well-Being [Negative Affect]* | *(Anglim et al., 2020) ** | 55495 | .56 | .65 | Original Data |
| Neuroticism & Well-Being [Negative Affect] | (DeNeve and Cooper, 1998) | 1092 | -.09 | - | Original Data |
| Neuroticism & Well-Being [Negative Affect] | (Joseph et al., 2015a) | 4457 | - | .70 | From (Watson et al., 1999) |
| Neuroticism & Well-Being [Negative Affect] | (Steel et al., 2008) | 16764 | .54 | .64 | Original Data |
| *Neuroticism & Well-Being [Positive Affect]* | *(Anglim et al., 2020) ** | 54816 | -.34 | -.39 | Original Data |
| Neuroticism & Well-Being [Positive Affect] | (Joseph et al., 2015a) | 4457 | - | -.40 | From (Watson et al., 1999) |
| Neuroticism & Well-Being [Positive Affect] | (Steel et al., 2008) | 11788 | -.30 | -.35 | Original Data |
| *Ability EI & Trait EI* | *(Joseph and Newman, 2010) *** | 1572 | .23 | .26 | Original Data |
| Ability EI & Academic Achievement | (MacCann et al., 2020b) | 5515 | .11 | .18 | Original Data |
| Ability EI & Academic Achievement | *(Somaa et al., 2021) ** | 5625 | .12 | .19 | Original Data |
| *Ability EI & Cognitive Ability* | *(Joseph and Newman, 2010) *** | 5538 | .22 | .25 | Original Data |
| Ability EI & Cognitive Ability | (O'Boyle et al., 2011) | 5192 | .26 | .32 | Original Data |
| Ability EI & Machiavellianism | (Miao et al., 2019) | 1419 | -.23 | -.31 | Original Data |
| *Ability EI & Machiavellianism* | *(Michels and Schulze, 2021) *** | 1561 | -.23 | -.31 | Original Data |
| Ability EI &  Narcissism | (Miao et al., 2019) | 1621 | -.09 | -.10 | Original Data |
| *Ability EI &*  *Narcissism* | *(Michels and Schulze, 2021) *** | 1859 | -.13 | -.16 | Original Data |
| Ability EI &  Narcissism | (Nguyen et al., 2022) | 1229 | -.11 | -.14 | Original Data |
| Ability EI &  Psychopathy | (Miao et al., 2019) | 1934 | -.18 | -.21 | Original Data |
| *Ability EI &*  *Psychopathy* | *(Michels and Schulze, 2021) *** | 3079 | -.18 | -.23 | Original Data |
| *Ability EI &*  *Job Performance* | *(Dogru, 2022) ** | 5100 | .24 | .28 | Original Data |
| Ability EI &  Job Performance | (Joseph and Newman, 2010) | 887 | .16 | .18 | Original Data |
| Ability EI &  Job Performance | (Joseph et al., 2015b) | 1287 | .17 | .20 | Original Data |
| Ability EI &  Job Performance | (O'Boyle et al., 2011) | 700 | .21 | .24 | Original Data |
| *Ability EI &*  *Job Satisfaction* | *(Dogru, 2022) ** | 4761 | .21 | .24 | Original Data |
| Ability EI &  Job Satisfaction | (Miao et al., 2017) | 1927 | .07 | .08 | Original Data |
| *Ability EI &*  *Creativity* | *(Xu et al., 2019) *** | 1929 | .08 | *-* | Original Data |
| *Ability EI & Self-Efficacy* | *(Joseph et al., 2015b) ** | 709 | .30 | .36 | Original Data |
| *Ability EI & Well-Being [Negative Affect]* | *(MacCann et al., 2020a) ** | 2290 | -.18 | -.21 | Original Data |
| *Ability EI &* Well-Being [Negative Affect] | (Miao et al., 2017) | 373 | -.32 | -.39 | Original Data |
| *Ability EI & Well-Being [Positive Affect]* | *(MacCann et al., 2020a) ** | 1895 | .04 | .05 | Original Data |
| Ability EI & Well-Being [Positive Affect] | (Miao et al., 2017) | 373 | -.06 | -.07 | Original Data |
| *Trait EI & Academic Achievement* | *(Somaa et al., 2021) ** | 19517 | .10 | .19 | Original Data |
| Trait EI & Academic Achievement | (MacCann et al., 2020b) | 4676 | .08 | .10 | Original Data |
| *Trait EI & Honesty-Humility* | *(Michels and Schulze, 2021) ** | 4119 | .10 | .12 | Original Data |
| *Trait EI & Cognitive Ability* | *(O'Boyle et al., 2011) *** | 5382 | .05 | .06 | Original Data |
| Trait EI & Machiavellianism | (Miao et al., 2019) | 4572 | -.22 | -.27 | Original Data |
| *Trait EI & Machiavellianism* | *(Michels and Schulze, 2021) *** | 6614 | -.23 | -.29 | Original Data |
| Trait EI &  Narcissism | (Miao et al., 2019) | 4612 | .04 | .05 | Original Data |
| *Trait EI &*  *Narcissism* | *(Michels and Schulze, 2021) *** | 6824 | .15 | .18 | Original Data |
| Trait EI &  Narcissism | (Nguyen et al., 2022) | 3027 | .21 | .26 | Original Data |
| Trait EI &  Psychopathy | (Miao et al., 2019) | 4785 | -.13 | -.16 | Original Data |
| *Trait EI &*  *Psychopathy* | *(Michels and Schulze, 2021) *** | 8127 | -.22 | -.25 | Original Data |
| *Trait EI &*  *Job Performance* | *(Dogru, 2022) ** | 10438 | .28 | .33 | Original Data |
| Trait EI &  Job Performance | (Miao et al., 2021) | 3086 | .46 | .54 | Original Data |
| Trait EI &  Job Performance | (O'Boyle et al., 2011) | 3961 | .24 | .28 | Original Data |
| Trait EI &  Job Satisfaction | (Dogru, 2022) | 8278 | .27 | .31 | Original Data |
| *Trait EI &*  *Job Satisfaction* | *(Miao et al., 2017) ** | 20116 | .28 | .32 | Original Data |
| *Trait EI &*  *Creativity* | *(Xu et al., 2019) *** | 15191 | .35 | - | Original Data |
| *Trait EI & Well-Being [Negative Affect]* | *(Miao et al., 2017) ** | 889 | -.36 | -.42 | Original Data |
| *Trait EI & Well-Being [Positive Affect]* | *(Miao et al., 2017) ** | 889 | .42 | .47 | Original Data |
| Academic Achievement & Cognitive Ability | (Kuncel et al., 2004) | 11368 | .27 | .39 | Original Data |
| *Academic Achievement & Cognitive Ability* | *(Poropat, 2009) ** | 17588 | - | .23 | Original Data |
| Academic Achievement & Cognitive Ability | (Richardson et al., 2012) | 7820 | .20 | .21 | Original Data |
| *Academic Achievement & Job Performance* | *(Roth et al., 1996) ** | 13984 | .16 | .23 | Original Data |
| *Academic Achievement & Creativity* | *(Gajda et al., 2017) ** | 11602 | .17 | *-* | Original Data |
| Academic Achievement & Self-Efficacy | (Credé and Philips, 2011) | 3798 | .18 | .21 | Original Data |
| *Academic Achievement & Self-Efficacy* | *(Richardson et al., 2012) ** | 46570 | .31 | .28 | Original Data |
| Academic Achievement & Self-Efficacy | (Richardson et al., 2012) | 1348 | .59 | .67 | Original Data |
| Academic Achievement & Self-Efficacy | (Robbins et al., 2004) | 9598 | .38 | .50 | Original Data |
| *Honesty-Humility & Cognitive Ability* | *(Lee et al., 2019) ** | 1833 | .04 | .05 | Original Data |
| Honesty-Humility & Machiavellianism | (Howard and van Zandt, 2020) | 18299 | -.54 | -.69 | Original Data |
| Honesty-Humility & Machiavellianism | (Muris et al., 2017) | 2227 | -.61 | - | Original Data |
| *Honesty-Humility & Machiavellianism* | *(Schreiber and Marcus, 2020) ** | 40818 | -.53 | ***-*** | Original Data |
| Honesty-Humility & Narcissism | (Howard and van Zandt, 2020) | 17981 | -.43 | -.56 | Original Data |
| Honesty-Humility & Narcissism | (Muris et al., 2017) | 2227 | -.41 | - | Original Data |
| *Honesty-Humility & Narcissism* | *(Schreiber and Marcus, 2020) ** | 40818 | -.42 | - | Original Data |
| Honesty-Humility & Psychopathy | (Howard and van Zandt, 2020) | 21114 | -.49 | -.63 | Original Data |
| Honesty-Humility & Psychopathy | (Muris et al., 2017) | 2227 | -.54 | - | Original Data |
| *Honesty-Humility & Psychopathy* | *(Schreiber and Marcus, 2020) ** | 40818 | -.47 | *-* | Original Data |
| *Honesty-Humility & Job Performance* | *(Lee et al., 2019) ** | 1161 | .10 | .15 | Original Data |
| *Honesty-Humility & Well-Being [Negative Affect]* | *(Anglim et al., 2020) ** | 4134 | -.15 | -.18 | Original Data |
| *Honesty-Humility & Well-Being [Positive Affect]* | *(Anglim et al., 2020) ** | 3834 | .07 | .09 | Original Data |
| *Psychoticism & Psychopathy* | *(Lilienfeld et al., 2014) ** | 3137 | .23 | - | Original Data |
| *Psychoticism & Job Satisfaction* | *(Steel et al., 2018) ** | 3078 | -.02 | -.03 | Original Data |
| *Psychoticism & Creativity* | *(Acar and Runco, 2012) ** | 6771 | .16 | *-* | Original Data |
| *Psychoticism &*  *Well-Being [Negative Affect]* | *(Steel et al., 2008) ** | 8867 | .03 | .04 | Original Data |
| *Psychoticism &*  *Well-Being [Positive Affect]* | *(Steel et al., 2008) ** | 8929 | .00 | .00 | Original Data |
| Cognitive Ability & Machiavellianism | (Michels, 2022) | 337 | .08 | .08 | Original Data |
| *Cognitive Ability & Machiavellianism* | *(O'Boyle et al., 2013) **** | *5904* | *.03* | *-* | Inputted Meta-Analysis |
| Cognitive Ability & Narcissism | (Michels, 2022) | 168 | .05 | .05 | Original Data |
| *Cognitive Ability & Narcissism* | *(O'Boyle et al., 2013) **** | 2727 | .01 | *-* | Inputted Meta-Analysis |
| *Cognitive Ability & Psychopathy* | *(Michels, 2022) *** | 19129 | -.07 | -.06 | Original Data |
| Cognitive Ability & Psychopathy | (O'Boyle et al., 2013) | 2950 | -.05 | - | Inputted Meta-Analysis |
| *Cognitive Ability & Cultural Intelligence* | *(Schlaegel et al., 2021) ** | 1460 | .07 | .07 | Original Data |
| Cognitive Ability & Job Performance | (Bertua et al., 2005) | 2469 | .22 | .48 | Original Data |
| Cognitive Ability & Job Performance | (Brown et al., 2011) | 10888 | - | .35 | From (Bobko et al., 1999) |
| Cognitive Ability & Job Performance | (Hülsheger et al., 2007) | 746 | .33 | .53 | Original Data |
| Cognitive Ability & Job Performance | (Joseph and Newman, 2010) | 32124 | .26 | .44 | From (Hunter and Hunter, 1984) |
| Cognitive Ability & Job Performance | (Kuncel et al., 2004) | 598 | .26 | .41 | Original Data |
| *Cognitive Ability & Job Performance* | *(Meriac et al., 2008) ** | 38350 | .30 | .34 | Original Data |
| Cognitive Ability & Job Performance | (van Iddekinge et al., 2017) | 4718 | .18 | .31 | Original Data |
| Cognitive Ability & Job Performance | (Vinchur et al., 1998) | 1770 | .18 | .31 | Original Data |
| *Cognitive Ability & Job Satisfaction* | *(Gonzalez-Mulé et al., 2017) ** | 29971 | .04 | .05 | Original Data |
| *Cognitive Ability & Creativity* | *(Serban et al., 2023) **** | 65829 | .27 | .33 | Inputted Meta-Analysis |
| Cognitive Ability & Creativity | (Kim, 2005) | 45880 | .17 | - | Original Data (Repeated Search) |
| Cognitive Ability & Self-Efficacy | (Badura et al., 2020) | 4426 | - | .02 | Original Data |
| *Cognitive Ability & Self-Efficacy* | *(Brown et al., 2011) ** | 5948 | - | .18 | From (Chen et al., 2001) |
| Cognitive Ability & Self-Efficacy | (Joseph et al., 2015b) | 4085 | .07 | .09 | Original Data |
| Cognitive Ability & Self-Efficacy | (Judge et al., 2007) | 4578 | - | .20 | Original Data |
| *Machiavellianism & Narcissism* | *(Muris et al., 2017) *** | 42359 | .34 | *-* | Original Data |
| Machiavellianism & Narcissism | (O'Boyle et al., 2012) | 8423 | .23 | .30 | Original Data |
| Machiavellianism & Narcissism | (Schreiber and Marcus, 2020) | 40818 | .38 | - | Original Data |
| *Machiavellianism & Psychopathy* | *(Muris et al., 2017) *** | 42359 | .58 | - | Original Data |
| Machiavellianism & Psychopathy | (O'Boyle et al., 2012) | 5762 | .46 | .59 | Original Data |
| Machiavellianism & Psychopathy | (Schreiber and Marcus, 2020) | 40818 | .54 | - | Original Data |
| *Machiavellianism & Job Performance* | *(O'Boyle et al., 2012) ** | 9297 | -.06 | -.07 | Original Data |
| *Machiavellianism & Job Satisfaction* | *(Bruk-Lee et al., 2009) ** | 1241 | -.19 | -.26 | Original Data |
| *Machiavellianism & Creativity* | *(Lebuda et al., 2021) *** | 10556 | .06 | - | Original Data |
| Narcissism & Psychopathy | (O'Boyle et al., 2012) | 8538 | .42 | .51 | Original Data |
| *Narcissism & Psychopathy* | *(Muris et al., 2017) *** | 42359 | .38 | - | Original Data |
| Narcissism & Psychopathy | (Schreiber and Marcus, 2020) | 40818 | .37 | - | Original Data |
| *Narcissism* & *Job Performance* | *(O'Boyle et al., 2012) ** | 3124 | -.02 | -.03 | Original Data |
| Narcissism & *Job Satisfaction* | *(Bruk-Lee et al., 2009) ** | 789 | -.07 | -.14 | Original Data |
| *Narcissism* & *Creativity* | *(Lebuda et al., 2021) *** | 12692 | .15 | - | Original Data |
| *Psychopathy & Job Performance* | *(O'Boyle et al., 2012) ** | 10227 | -.08 | -.10 | Original Data |
| *Psychopathy & Creativity* | *(Lebuda et al., 2021) *** | 11053 | .03 | - | Original Data |
| *Cultural Intelligence &* *Job Performance* | *(Schlaegel et al., 2021) ** | 5884 | .37 | .47 | Original Data |
| *Cultural Intelligence &* *Job Satisfaction* | *(Schlaegel et al., 2021) ** | 1278 | .27 | .36 | Original Data |
| Job Performance & Job Satisfaction | (Bowling et al., 2015) | 19494 | .20 | .27 | Original Data |
| *Job Performance & Job Satisfaction* | *(Judge et al., 2001) ** | 54471 | .18 | .30 | Original Data |
| Job Performance & Self-Efficacy | (Joseph et al., 2015b) | 2703 | .10 | .13 | Original Data |
| Job Performance & Self-Efficacy | (Judge and Bono, 2001) | 1122 | .19 | .23 | Original Data |
| *Job Performance & Self-Efficacy* | *(Judge et al., 2007) ** | 32123 | - | .37 | Original Data |
| Job Performance & Self-Efficacy | (Stajkovic and Luthans, 1998) | 21616 | .38 | .34 | Original Data |
| Job Performance & Well-Being [Negative Affect] | (Gutiérrez et al., 2020) | 127 | -.16 | -.16 | Original Data |
| *Job Performance & Well-Being [Negative Affect]* | *(Kaplan et al., 2009) ** | 7164 | -.15 | ***-*** | Original Data |
| *Job Performance & Well-Being [Positive Affect]* | *(Gutiérrez et al., 2020) ** | 11446 | .36 | .37 | Original Data |
| Job Performance & Well-Being [Positive Affect] | (Kaplan et al., 2009) | 3084 | .19 | - | Original Data |
| *Job Satisfaction &* *Creativity* | *(Ouyang et al., 2021) ** | 1442 | .40 | .44 | Original Data |
| *Job Satisfaction &* *Self-Efficacy* | *(Ahrari et al., 2021) ** | 19007 | - | .42 | Original Data |
| Job Satisfaction & Self-Efficacy | (Judge and Bono, 2001) | 12903 | .38 | .45 | Original Data |
| Job Satisfaction & Well-Being [Negative Affect] | (Bowling et al., 2008) | 3100 | -.25 | -.29 | Original Data |
| Job Satisfaction & Well-Being [Negative Affect] | (Bowling et al., 2010) | 15662 | -.27 | -.32 | Original Data |
| Job Satisfaction & Well-Being [Negative Affect] | (Bruk-Lee et al., 2009) | 12834 | -.25 | -.30 | Original Data |
| *Job Satisfaction & Well-Being [Negative Affect]* | *(Kaplan et al., 2009) ** | 52120 | -.37 | - | From (Thoresen et al., 2003) |
| Job Satisfaction & Well-Being [Positive Affect] | (Bowling et al., 2008) | 2174 | .45 | .52 | Original Data |
| Job Satisfaction & Well-Being [Positive Affect] | (Bowling et al., 2010) | 12343 | .38 | .44 | Original Data |
| Job Satisfaction & Well-Being [Positive Affect] | (Bruk-Lee et al., 2009) | 6595 | .41 | .40 | Original Data |
| *Job Satisfaction & Well-Being [Positive Affect]* | *(Kaplan et al., 2009) ** | 22148 | .33 | - | From (Thoresen et al., 2003) |
| Creativity & Self-Efficacy | (Haase et al., 2018) | 17226 | .39 | .50 | Original Data |
| Creativity & Self-Efficacy | (Koh et al., 2018) | 7160 | .43 | .50 | Original Data |
| *Creativity & Self-Efficacy* | *(Liu et al., 2016) ** | 19973 | .35 | .40 | Original Data |
| Creativity & Self-Efficacy | (Ng and Feldman, 2012) | 1145 | .45 | - | Original Data |
| Creativity & Self-Efficacy | (Ouyang et al., 2021) | 2183 | .47 | .55 | Original Data |
| Creativity & Well-Being [Positive Affect] | (Ng and Feldman, 2012) | 965 | .27 | - | Original Data |
| *Creativity & Well-Being [Positive Affect]* | *(Ouyang et al., 2021) ** | 1447 | .45 | .54 | Original Data |
| Well-Being [Negative Affect] & *&* Well-Being [Positive Affect] | (Anglim et al., 2020) | 903 | -.39 | - | Original Data |
| *Well-Being [Negative Affect] & & Well-Being [Positive Affect]* | *(Joseph et al., 2015a) ** | 4457 | - | -.22 | From (Watson et al., 1999) |

*Note.* * - used as master correlation for a given construct pairing;

** - used as master correlation within input matrix;

*** - meta-analyses used as master correlations but that were not obtained in the original search.

## **Table S4**

*List of Assessments Used to Operationalize Constructs in the Meta-Meta-Analysis*

| Constructs | Assessment operationalizations |
| --- | --- |
| 1-5. Openness; Conscientiousness; Extraversion; Agreeableness; & Neuroticism | 16PF (Sixteen Personality Factor Questionnaire)  ABOS (Attitudinal and Behavioral Openness Scale)  BFAS (Big Five Aspect Scales)  BFI (Big Five Inventory)  BFI-10 (Big Five Inventory 10-Item)  FDTI (Five-Dimensional Temperament Inventory  HPI (Hamburg Personality Inventory)  HPI (Hogan Personality Inventory)  IPIP (International Personality Item Pool)  NEO-FFI (NEO Five Factor Inventory)  NEO-PI (NEO Personality Inventory)  NEO-PI-R (NEO Personality Inventory Revised)  OPQ (Occupational Personality Questionnaire)  PCI (Personal Characteristics Inventory)  PSI (Personal Style Inventory)  SIMP (Single Item Personality Pool)  TDA (Trait Descriptive Adjective Scale)  TIPI (Ten Item Personality Inventory) |
| 6. Ability EI (PB) | EIS (Emotional Intelligence Scale)  EMAT (Emotional Management Ability Test)  MEIS (Multifactor Emotional Intelligence Scale)  MSCEIT (Mayer-Salovey-Caruso Emotional Intelligence Test)  RTME (Reading the Mind in the Eyes Task)  STEM (Situational Test of Emotion Management)  STEU (Situational Test of Emotion Understanding)  WEIP (Work Group Emotional Intelligence Profile) |
| 7. Trait EI | EIQ (Emotional Intelligence Questionnaire)  Emotional Quotient Index  ERC (Emotion Regulation Checklist)  EQ-i (Emotional Quotient Inventory)  Genos  MMEI (Multidimensional Measure of Emotional Intelligence)  TEIQue (Trait Emotional Intelligence Questionnaire)  TEIQue-SF (Trait Emotional Intelligence Questionnaire Short Form)  WLEIS (Wong and Law Emotional Intelligence Scale) |
| 8. Cognitive Ability | ACT (American College Testing)  AGCT (Army General Classification Test)  ASVAB (Armed Services Vocational Aptitude Battery)  AUT (Alternate Uses Test)  Culture Fair  GZAT (Guilford-Zimmerman Abilities Test)  OST (Object-Sorting Task)  Otis-Lennon Test of Mental Maturity  OQS (Otis Quick Scoring Mental Ability Test)  QWT (Quick Word Test)  Raven’s Progressive Matrices  SAT  SILS (Shipley Institute of Living Scale)  WA-R&W (Wollack Alert Reading and Writing)  WAIS-III (Wechsler Adult Intelligence Scale–III)  WAIS-R (Wechsler Adult Intelligence Scale—Revised)  Wonderlic |
| 9. Machiavellianism | DD (Dirty Dozen)  MACH-IV  MACH-V  SD3 (Short Dark Triad) |
| 10. Narcissism (Grandiose) | DD (Dirty Dozen)  Nach-C  Nach-E  NPI (Narcissistic Personality Inventory)  Psychological Entitlement Scale  SD3/SDT (Short Dark Triad)  State-Trait Grandiosity Scale  Supernumerary Personality Inventory  Wink-Gough Narcissism Scale |
| 11. Psychopathy | DD (Dirty Dozen)  PCL (Psychopathy Checklist)  PPI (Psychopathic Personality Inventory)  SD3 (Short Dark Triad)  SRP-III (Self-Report of Psychopathy, Third Revision) |
| 12. Creativity | ASAS (Artistic and Scientific Activities Survey)  AUT (Alternative Uses Task)  CAQ (Creative Achievement Questionnaire)  CPI (Creative Personal Identity)  CPS (Creative Personality Scale)  CSDD (Creativity Scale for Different Domains)  CDQ (Creativity Domain Questionnaire)  CSE (Creative Self-Efficacy)  CSP (Creative Self-Perception)  CVT (Creative Visualization Task)  K-DOCS (Kaufman Domains of Creativity Scale)  RAT (Remote Associate Test)  RIBS (Runco Ideational Behavior Scale)  SEC (Self-Estimated Creativity)  SRC (Self-Rated Creativity)  TCT (Test for Creative Thinking)  TICT (Torrance Test of Creative Thinking)  WCAT (Williams Creative Aptitude Test) |

# **References**

Acar, S. & Runco, M. A. 2012. Psychoticism and Creativity: A Meta-analytic Review. *Psychology of Aesthetics, Creativity, and the Arts,* 6**,** 341-350.

Ackerman, P. L. & Heggestad, E. D. 1997. Intelligence, personality, and interests: Evidence for overlapping traits. *Psychological Bulletin,* 121**,** 219-245.

Ahrari, S., Roslan, S., Zaremohzzabieh, Z., Rasdi, R. M. & Abu Samah, A. 2021. Relationship between teacher empowerment and job satisfaction: A Meta-Analytic path analysis. *Cogent Education,* 8.

Akpur, U. 2020. A Systematic Review and Meta-Analysis on the Relationship Between Emotional Intelligence and Academic Achievement. *Kuram ve Uygulamada Egitim Bilimleri,* 20**,** 51-64.

Alabbasi, A. M. A., Ayoub, A. E. A. & Ziegler, A. 2021. Are gifted students more emotionally intelligent than their non-gifted peers? A meta-analysis. *High Ability Studies,* 32.

Anglim, J., Dunlop, P. D., Wee, S., Horwood, S., Wood, J. K. & Marty, A. 2022. Personality and intelligence: A meta-analysis. *Psychological Bulletin,* 148**,** 301–336.

Anglim, J., Horwood, S., Smilie, L. D., Marrero, R. J. & Wood, J. K. 2020. Predicting Psychological and Subjective Well-Being From Personality: A Meta-Analysis. *Psychological Bulletin,* 146**,** 279-323.

Badura, K. L., Grijalva, E., Galvin, B. M., Owens, B. P. & Joseph, D. L. 2020. Motivation to lead: A meta-analysis and distal-proximal model of motivation and leadership. *Journal of Applied Psychology,* 105**,** 331-354.

Barrett, G. V., Miguel, R. F., Hurd, J. M., Lueke, S. B. & Tan, J. A. 2003. Practical issues in the use of personality tests in police selection. *Public Personnel Management,* 32.

Barrick, M. R. & Mount, M. K. 1991. The Big Five personality dimensions and job performance: A meta-analysis. *Personnel Psychology,* 44**,** 1-26.

Barrick, M. R., Mount, M. K. & Judge, T. A. 2001. Personality and performance at the beginning of the new millennium: What do we know and where do we go next? *International Journal of Selection and Assessment,* 9**,** 9-30.

Bertua, C., Anderson, N. & Salgado, J. F. 2005. The predictive validity of cognitive ability tests: A UK meta-analysis. *Journal of Occupational and Organizational Psychology,* 78**,** 387-409.

Bobko, P., Roth, P. L. & Potosky, D. 1999. Derivation and implications of a meta-analytic incorporating cognitive ability, alternative predictors, and job performance. *Personnel Psychology,* 52**,** 561-589.

Bowling, N. A. 2007. Is the job satisfaction–job performance relationship spurious? A meta-analytic examination. *Journal of Vocational Behavior,* 71**,** 167-185.

Bowling, N. A., Eschleman, K. J. & Wang, Q. 2010. A meta-analytic examination of the relationship between job satisfaction and subjective well-being. *Journal of Occupational and Organizational Psychology,* 83**,** 915-934.

Bowling, N. A., Hendricks, E. A. & Wagner, S. H. 2008. Positive and Negative Affectivity and Facet Satisfaction: A Meta-analysis. *Journal of Business and Psychology,* 23**,** 115-125.

Bowling, N. A., Khazon, S., Meyer, R. D. & Burrus, C. J. 2015. Situational strength as a moderator of the relationship between job satisfaction and job performance: A meta-analytic examination. *Journal of Business and Psychology,* 30**,** 89-104.

Brown, S. D., Lent, R. W., Telander, K. & Tramayne, S. 2011. Social cognitive career theory, conscientiousness, and work performance: A meta-analytic path analysis. *Journal of Vocational Behavior,* 79**,** 81-90.

Bruk-Lee, V., Khoury, H. A., Nixon, A. E., Goh, A. & Spector, P. E. 2009. Replicating and Extending Past Personality/Job Satisfaction Meta-Analyses. *Human Performance,* 22.

Cerni, T., Di Benedetto, A. & Rumiati, R. I. 2021. The Contribution of Personality and Intelligence Toward Cognitive Competences in Higher Education. *Frontiers In Psychology,* 12.

Chen, G., Casper, W. J. & Cortina, J. M. 2001. The roles of self-efficacy and task complexity in the relationships among cognitive ability, conscientiousness, and work-related performance: A meta-analytic examination. *Human Performance,* 14.

Connelly, B. S. & Ones, D. S. 2010. An other perspective on personality: meta-analytic integration of observers' accuracy and predictive validity. *Psychological Bulletin,* 136**,** 1092-1122.

Cortina, J. M., Goldstein, N. B., Payne, S. C., Davison, H. K. & Gilliland, S. W. 2000. The incremental validity of interview scores over and above cognitive ability and conscientiousness scores. *Personnel Psychology,* 53**,** 325-351.

Credé, M. & Philips, L. A. 2011. A meta-analytic review of the Motivated Strategies for Learning Questionnaire. *Learning and Individual Differences,* 21**,** 337-346.

Cuadrado, D., Salgado, J. F. & Moscoso, S. 2021. Personality, Intelligence, and Counterproductive Academic Behaviors: A Meta-Analysis. *Journal of Personality and Social Psychology,* 120**,** 504-537.

Darr, W. 2011. Military Personality Research: A Meta-Analysis of the Self Description Inventory. *Military Psychology,* 23**,** 272-296.

DeNeve, K. M. & Cooper, H. 1998. The happy personality: A meta-analysis of 137 personality traits and subjective well-being. *Psychological Bulletin,* 124**,** 197-229.

Diedrich, J., Jauk, E., Silvia, P. J., Gredlein, J. M., Neubauer, A. C. & Benedek, M. 2018. Assessment of real-life creativity: The Inventory of Creative Activities and Achievements (ICAA). *Psychology of Aesthetics, Creativity, and the Arts,* 12**,** 304-316.

Do, M. H. & Minbashian, A. 2014. A meta-analytic examination of the effects of the agentic and affiliative aspects of extraversion on leadership outcomes. *The Leadership Quarterly,* 25**,** 1040-1053.

Doan, T., Kanjanakan, P., Zhu, D. & Kim, P. B. 2021. Consequences of employee personality in the hospitality context: a systematic review and meta-analysis. *International Journal of Contemporary Hospitality Management,* 33.

Dogru, C. 2022. A Meta-Analysis of the Relationships Between Emotional Intelligence and Employee Outcomes. *Frontiers in Psychology,* 13.

Gajda, A., Karwowski, M. & Beghetto, R. A. 2017. Creativity and Academic Achievement: A Meta-Analysis. *Journal of Educational Psychology,* 109**,** 269-299.

Gatzka, T. & Hell, B. 2018. Openness and Postsecondary Academic Performance: A Meta-Analysis of Facet-, Aspect-, and Dimension-Level Correlations. *Journal of Educational Psychology,* 110**,** 355-377.

Gignac, G. E. 2021. People who consider themselves smart do not consider themselves interpersonally challenged: Convergent validity evidence for subjectively measured IQ and EI. *Personality and Individual Differences,* 174.

Gong, Z. & Jiao, X. 2019. Are Effect Sizes in Emotional Intelligence Field Declining? A Meta-Meta Analysis. *Frontiers in Psychology,* 10.

Gonzalez-Mulé, E., Carter, K. M. & Mount, M. K. 2017. Are smarter people happier? Meta-analyses of the relationships between general mental ability and job and life satisfaction. *Journal of Vocational Behavior,* 99**,** 146-164.

Grijalva, E., Harms, P. D., Newman, D. A., Gaddis, B. H. & Fraley, C. R. 2015. Narcissism and Leadership: A Meta-Analytic Review of Linear and Nonlinear Relationships. *Personnel Psychology,* 68**,** 1-47.

Grijalva, E. & Newman, D. A. 2014. Narcissism and Counterproductive Work Behavior (CWB): Meta-Analysis and Consideration of Collectivist Culture, Big Five Personality, and Narcissism's Facet Structure. *Applied Psychology,* 64**,** 93-126.

Gutiérrez-Cobo, M. J., Cabello González, R. & Fernández-Berrocal, P. 2016. The Relationship between Emotional Intelligence and Cool and Hot Cognitive Processes: A Systematic Review *Frontiers in Behavioral Neuroscience,* 10.

Gutiérrez, O. I., Polo, J. D., Zambrano, M. J. & Molina, D. C. 2020. Meta-analysis and Scientific Mapping of Well-being and Job Performance. *The Spanish Journal of Psychology,* 23.

Haase, J., Hoff, E. V., Hanel, P. H. P. & Innes-Ker, Å. 2018. A Meta-Analysis of the Relation between Creative Self-Efficacy and Different Creativity Measurements. *Creativity Research Journal,* 30**,** 1-16.

He, Y., Donnellan, B. M. & Mendoza, A. M. 2019. Five-factor personality domains and job performance: A second order meta-analysis. *Journal of Research in Personality,* 82.

Hough, L. M. 1992. The 'Big Five' Personality Variables--Construct Confusion: Description Versus Prediction. *Human Performance,* 5**,** 139-155.

Howard, M. C. & van Zandt, E. C. 2020. The discriminant validity of honesty-humility: A meta-analysis of the HEXACO, Big Five, and Dark Triad. *Journal of Research in Personality,* 87.

Hülsheger, U. R., Maier, G. W. & Stumpp, T. 2007. Validity of general mental ability for the prediction of job performance and training success in Germany: A meta-analysis. *International Journal of Selection and Assessment,* 15**,** 3-18.

Hunter, J. E. & Hunter, R. F. 1984. Validity and utility of alternative predictors of job performance. *Psychological Bulletin,* 96**,** 72-98.

Hurtz, G. M. & Donovan, J. J. 2000. Personality and job performance: The Big Five revisited. *Journal of Applied Psychology,* 85**,** 869-879.

Joseph, D. L., Dhanani, L. Y., Shen, W., McHugh, B. C. & McCord, M. A. 2015a. Is a happy leader a good leader? A meta-analytic investigation of leader trait affect and leadership. *The Leadership Quarterly,* 26**,** 557-576.

Joseph, D. L., Jin, J., Newman, D. A. & O'Boyle, E. H. 2015b. Why does self-reported emotional intelligence predict job performance? A meta-analytic investigation of mixed EI. *Journal of Applied Psychology,* 100**,** 298-342.

Joseph, D. L. & Newman, D. A. 2010. Emotional intelligence: An integrative meta-analysis and cascading model. *Journal of Applied Psychology,* 95**,** 54-78.

Judge, T. A. & Bono, J. E. 2001. Relationship of core self-evaluations traits-self-esteem, generalized self-efficacy, locus of control, and emotional stability-with job satisfaction and job performance: A meta-analysis. *Journal of Applied Psychology,* 86**,** 80-92.

Judge, T. A., Erez, A., Bono, J. E. & Thoresen, C. J. 2002a. Are measures of self-esteem, neuroticism, locus of control, and generalized self-efficacy indicators of a common core construct? *Journal of Personality and Social Psychology,* 83**,** 693-710.

Judge, T. A., Heller, D. & Mount, M. K. 2002b. Five-factor model of personality and job satisfaction: A meta-analysis. *Journal of Applied Psychology,* 87**,** 530-541.

Judge, T. A. & Ilies, R. 2002. Relationship of personality to performance motivation: A meta-analytic review. *Journal of Applied Psychology,* 87**,** 797-807.

Judge, T. A., Jackson, C. L., Shaw, J. C., Scott, B. A. & Rich, B. L. 2007. Self-efficacy and work-related performance: The integral role of individual differences. *Journal of Applied Psychology,* 92**,** 107-127.

Judge, T. A., Thoresen, C. J., Bono, J. E. & Patton, G. K. 2001. The job satisfaction-job performance relationship: A qualitative and quantitative review. *Psychological Bulletin,* 127**,** 376-407.

Kaplan, S., Bradley, J. C., Luchman, J. N. & Haynes, D. 2009. On the Role of Positive and Negative Affectivity in Job Performance: A Meta-Analytic Investigation. *Journal of Applied Psychology,* 94**,** 162-176.

Karwowski, M. & Lebuda, I. 2015. The Big Five, the Huge Two, and Creative Self-Beliefs: A Meta-Analysis. *Psychology of Aesthetics, Creativity, and the Arts,* 10**,** 214-232.

Kepes, S. & McDaniel, M. A. 2015. The Validity of Conscientiousness Is Overestimated in the Prediction of Job Performance. *PLoS One,* 10.

Kim, K. H. 2005. Can Only Intelligent People Be Creative? A Meta-Analysis. *Journal of Advanced Academics,* 16.

Koh, D., Lee, K. & Joshi, K. 2018. Transformational leadership and creativity: A meta-analytic review and identification of an integrated model. *Journal of Organizational Behavior,* 40**,** 625-650.

Kuncel, N. R., Hezlett, S. A. & Ones, D. S. 2004. Academic performance, career potential, creativity, and job performance: Can one construct predict them all? *Journal of Personality and Social Psychology,* 86**,** 148-161.

Lebuda, I., Figura, B. & Karwowski, M. 2021. Creativity and the Dark Triad: A meta-analysis. *Journal of Research in Personality,* 92.

Lee, Y., Berry, C. M. & Gonzalez-Mulé, E. 2019. The Importance of Being Humble: A Meta-Analysis and Incremental Validity Analysis of the Relationship Between Honesty-Humility and Job Performance. *Journal of Applied Psychology,* 104**,** 1535-1546.

Lilienfeld, S. O., Watts, A. L., Smith, S. F., Berg, J. M. & Latzman, R. D. 2014. Psychopathy Deconstructed and Reconstructed: Identifying and Assembling the Personality Building Blocks of Cleckley's Chimera. *Journal of Personality,* 83**,** 593-610.

Liu, D., Jiang, K., Shalley, C. E., Keem, S. & Zhou, J. 2016. Motivational mechanisms of employee creativity: A meta-analytic examination and theoretical extension of the creativity literature. *Organizational Behavior and Human Decision Processes,* 137**,** 236–263.

MacCann, C., Erbas, Y., Dejonckheere, E., Minbashian, A., Kuppens, P. & Fayn, K. 2020a. Emotional Intelligence Relates to Emotions, Emotion Dynamics, and Emotion Complexity A Meta-Analysis and Experience Sampling Study. *European Journal of Psychological Assessment,* 36**,** 460-470.

MacCann, C., Jiang, Y., Brown, L. E. R., Double, K. S., Bucich, M. & Minbashian, A. 2020b. Emotional Intelligence Predicts Academic Performance: A Meta-Analysis. *Psychological Bulletin,* 146**,** 150-186.

Mammadov, S. 2022. Big Five personality traits and academic performance: A meta-analysis. *Journal of Personality,* 90**,** 222-255.

Mayer, J. D., Salovey, P. & Caruso, D. R. 2004. Emotional Intelligence: Theory, Findings, and Implications. *Psychological Inquiry,* 15**,** 197-215.

McAbee, S. T. & Oswald, F. L. 2013. The Criterion-Related Validity of Personality Measures for Predicting GPA: A Meta-Analytic Validity Competition. *Psychological Assesssment,* 25**,** 532-544.

Meriac, J. P., Hoffman, B. J., Woehr, D. J. & Fleisher, M. S. 2008. Further evidence for the validity of assessment center dimensions: A meta-analysis of the incremental criterion-related validity of dimension ratings. *Journal of Applied Psychology,* 93**,** 1042-1052.

Miao, C., Humphrey, R. H. & Qian, S. 2017. A meta-analysis of emotional intelligence and work attitudes. *Journal of Occupational and Organizational Psychology,* 90**,** 177-202.

Miao, C., Humphrey, R. H. & Qian, S. 2021. Emotional intelligence and job performance in the hospitality industry: a meta-analytic review. *International Journal of Contemporary Hospitality Management,* 33.

Miao, C., Humphrey, R. H., Qian, S. & Pollack, J. M. 2019. The relationship between emotional intelligence and the dark triad personality traits: A meta-analytic review. *Journal of Research in Personality,* 78**,** 189-197.

Michels, M. 2022. General Intelligence and the Dark Triad. *Journal of Individual Differences,* 43**,** 35–46.

Michels, M. & Schulze, R. 2021. Emotional intelligence and the dark triad: A meta-analysis. *Personality and Individual Differences,* 180.

Mol, S. T., Born, M. P., Willemsen, M. E. & van der Molen, H. T. 2005. Predicting expatriate job performance for selection purposes - A quantitative review. *Journal of Cross-Cultural Psychology,* 36.

Mount, M. K., Barrick, M. R. & Stewart, G. L. 1998. Five-Factor Model of personality and performance in jobs involving interpersonal interactions. *Human Performance,* 11.

Muris, P., Merckelbach, H., Otgaar, H. & Meijer, E. 2017. The malevolent side of human nature. *Perspectives on Psychological Science,* 12**,** 183–204.

Ng, T. W. H., Eby, L. T., Sorensen, K. L. & Feldman, D. C. 2005. Predictors of objective and subjective career success: A meta-analysis. *Personnel Psychology,* 58**,** 367-408.

Ng, T. W. H. & Feldman, D. C. 2012. A comparison of self-ratings and non-self-report measures of employee creativity. *Human Relations,* 65.

Nguyen, N. N., Takahashi, Y. & Nham, T. P. 2022. Relationship between emotional intelligence and narcissism: a meta-analysis. *Management Research Review,* 45**,** 1338-1353.

O'Boyle, E. H., Forsyth, D. R., Banks, G. C. & McDaniel, M. A. 2012. A Meta-Analysis of the Dark Triad and Work Behavior: A Social Exchange Perspective. *Journal of Applied Psychology,* 97**,** 557-579.

O'Boyle, E. H., Forsyth, D. R., Banks, G. C. & Story, P. A. 2013. A meta-analytic review of the Dark Triad–intelligence connection. *Journal of Research in Personality,* 47**,** 789–794.

O'Boyle, E. H., Forsyth, D. R., Banks, G. C., Story, P. A. & White, C. D. 2014. A Meta‐Analytic test of redundancy and relative importance of the Dark triad and Five‐Factor model of personality. *Journal of Personality,* 83**,** 644–664.

O'Boyle, E. H., Humphrey, R. H., Pollack, J. M., Hawver, T. H. & Story, P. A. 2011. The relation between emotional intelligence and job performance: A meta‐analysis. *Journal of Organizational Behavior,* 32**,** 788-818.

O'Connor, M. & Paunonen, S. V. 2007. Big five personality predictors of post-secondary academic performance. *Personality and Individual Differences,* 43**,** 971-990.

Ogurlu, U. 2021. A meta-analytic review of emotional intelligence in gifted individuals: a multilevel analysis. *Personality and Individual Differences,* 171.

Olderbak, S., Semmler, M. & Doebler, P. 2018. Four-Branch Model of Ability Emotional Intelligence with Fluid and Crystallized Intelligence: A Meta-Analysis of Relations. *Emotion Review,* 11.

Ouyang, X., Liu, Z. & Gui, C. 2021. Creativity in the hospitality and tourism industry: a meta-analysis. *International Journal of Contemporary Hospitality Management,* 33.

Perera, H. N. & DiGiacomo, M. 2013. The relationship of trait emotional intelligence with academic performance: A meta-analytic review. *Learning and Individual Differences,* 28**,** 20-33.

Poropat, A. E. 2009. A meta-analysis of the five-factor model of personality and academic performance. *Psychological Bulletin,* 135**,** 322-338.

Richardson, M., Abraham, C. & Bond, R. 2012. Psychological correlates of university students' academic performance: A systematic review and meta-analysis. *Psychological Bulletin,* 138**,** 353-387.

Robbins, S. B., Lauver, K., Le, H., Davis, D., Langley, R. & Carlstrom, A. 2004. Do Psychosocial and Study Skill Factors Predict College Outcomes? A Meta-Analysis. *Psychological Bulletin,* 130**,** 261-288.

Roth, P. L., BeVier, C. A., Switzer, F. S. I. & Schippmann, J. S. 1996. Meta-analyzing the relationship between grades and job performance. *Journal of Applied Psychology,* 81**,** 548-556.

Salgado, J. F. 1997. The five factor model of personality and job performance in the European Community. *Journal of Applied Psychology,* 82**,** 30-43.

Salgado, J. F. 1998. Big Five personality dimensions and job performance in army and civil occupations: A European perspective. *Human Performance,* 11.

Salgado, J. F. 2003. Predicting job performance using FFM and non-FFM personality measures. *Journal of Occupational and Organizational Psychology,* 76**,** 323-346.

Salgado, J. F. & Tauriz, G. 2012. The Five-Factor Model, forced-choice personality inventories and performance: A comprehensive meta-analysis of academic and occupational validity studies. *European Journal of Work and Organizational Psychology,* 23.

Sánchez-Álvarez, N., Berrios Martos, M. P. & Extremera, N. 2020. A Meta-Analysis of the Relationship Between Emotional Intelligence and Academic Performance in Secondary Education: A Multi-Stream Comparison. *Frontiers in Psychology,* 11.

Schilling, M., Becker, N., Grabenhorst, M. M. & Konig, C. J. 2020. The relationship between cognitive ability and personality scores in selection situations: A meta-analysis. *International Journal of Selection and Assessment,* 29**,** 1-18.

Schlaegel, C., Richter, N. F. & Taras, V. 2021. Cultural intelligence and work-related outcomes: A meta-analytic examination of joint effects and incremental predictive validity. *Journal of World Business,* 56.

Schreiber, A. & Marcus, B. 2020. The place of the “Dark Triad” in general models of personality: Some meta-analytic clarification. *Psychological Bulletin,* 146**,** 1021–1041.

Serban, A., Kepes, S., Wang, W. & Baldwin, R. 2023. Cognitive ability and creativity: Typology contributions and a meta-analytic review. *Intelligence,* 98.

Somaa, F., Asghar, A. & Hamid, P. F. 2021. Academic Performance and Emotional Intelligence with Age and Gender as Moderators: A Meta-analysis. *Developmental Neuropsychology,* 46.

Stajkovic, A. D. & Luthans, F. 1998. Self-efficacy and work-related performance: A meta-analysis. *Psychological Bulletin,* 124**,** 240-261.

Stankov, L. 2018. Low Correlations between Intelligence and Big Five Personality Traits: Need to Broaden the Domain of Personality. *Journal of Intelligence,* 6.

Steel, P., Schmidt, J., Bosco, F. & Uggerslev, K. 2018. The effects of personality on job satisfaction and life satisfaction: A meta-analytic investigation accounting for bandwidth-fidelity and commensurability. *Human Relations,* 72.

Steel, P., Schmidt, J. & Shultz, J. 2008. Refining the Relationship Between Personality and Subjective Well-Being. *Psychological Bulletin,* 134**,** 138-161.

Tett, R. P., Jackson, D. N. & Rothstein, M. 1991. Personality Measures as Predictors of Job-Performance - A Meta-Analytic Review. *Personnel Psychology,* 44**,** 703-742.

Thielmann, I., Moshagen, M., Hilbig, B. E. & Zettler, I. 2021. On the Comparability of Basic Personality Models: Meta-Analytic Correspondence, Scope, and Orthogonality of the Big Five and HEXACO Dimensions. *European Journal of Personality,* 36.

Thoresen, C. J., Kaplan, S. A., Barsky, A. P., Warren, C. R. & de Chermont, K. 2003. The affective underpinnings of job perceptions and attitudes: A meta-analytic review and integration. *Psychological Bulletin,* 129**,** 914-945.

Trapmann, S., Hell, B., Hirn, J.-O. W. & Schuler, H. 2007. Meta-Analysis of the Relationship Between the Big Five and Academic Success at University. *Journal of Psychology,* 215.

Trzesniewski, K. H., Donnellan, B. M. & Robins, R. W. 2008. Is “Generation Me” really more narcissistic than previous generations? *Journal of Personality,* 76**,** 903-918.

van Aarde, N., Meiring, D. & Wiernik, B. M. 2017. The validity of the Big Five personality traits for job performance: Meta-analyses of South African studies. *International Journal of Selection and Assessment,* 25**,** 223-239.

van der Linden, D., Pekaar, K. A., Bakker, A. B., Schermer, J. A., Vernon, P. A., Dunkel, C. S. & Petrides, K. V. 2017. Overlap between the general factor of personality and emotional intelligence: A meta-analysis. *Psychological Bulletin,* 143**,** 36-52.

van der Linden, D., te Nijenhuis, J. & Bakker, A. B. 2010. The General Factor of Personality: A meta-analysis of Big Five intercorrelations and a criterion-related validity study. *Journal of Research in Personality,* 44**,** 315-327.

van Iddekinge, C. H., Aguinis, H., Mackey, J. D. & DeOrtentiis, P. S. 2017. A Meta-Analysis of the Interactive, Additive, and Relative Effects of Cognitive Ability and Motivation on Performance. *Journal of Management,* 44.

van Rooy, D. L., Viswesvaran, C. & Pluta, P. 2005. An evaluation of construct validity: What is this thing called emotional intelligence? *Human Performance,* 18**,** 445-462.

Vashisht, S., Kaushal, P. & Vashisht, R. 2021. Emotional intelligence, Personality Variables and Career Adaptability: A Systematic Review and Meta-analysis. *Vision: The Journal of Business Perspective,* 27.

Vedel, A. 2014. The Big Five and tertiary academic performance: A systematic review and meta-analysis. *Personality and Individual Differences,* 71**,** 66-76.

Vinchur, A. J., Schippmann, J. S., Switzer, F. S. I. & Roth, P. L. 1998. A meta-analytic review of predictors of job performance for salespeople. *Journal of Applied Psychology,* 83**,** 586-597.

Vize, C. E., Lynam, D. R., Collison, K. L. & Miller, J. D. 2018. Differences Among Dark Triad Components: A Meta-Analytic Investigation. *Personality Disorders: Theory, Research, and Treatment,* 9**,** 101-111.

Watrin, L., Weihrauch, L. & Wilhelm, O. 2022. The criterion-related validity of conscientiousness in personnel selection: A meta-analytic reality check. *International Journal of Selection and Assessment,* 31**,** 286-301.

Watson, D., Wiese, D., Vaidya, J. & Tellegen, A. 1999. The two general activation systems of affect: Structural findings, evolutionary considerations, and psychobiological evidence. *Journal of Personality and Social Psychology,* 76**,** 820-838.

Wilmot, M. P., Wanberg, C. R., Kammeyer-Mueller, J. D. & Ones, D. S. 2019. Extraversion advantages at work: A quantitative review and synthesis of the meta-analytic evidence. *Journal of Applied Psychology,* 104**,** 1447–1470.

Wolf, M. B. & Ackerman, P. L. 2005. Extraversion and intelligence: A meta-analytic investigation. *Personality and Individual Differences,* 39**,** 531-542.

Woo, S. E., Chernyshenko, O. S., Stark, S. E. & Conz, G. 2013. Validity of Six Openness Facets in Predicting Work Behaviors: A Meta-Analysis. *Journal of Personality Assessment,* 96**,** 76–86.

Xu, X., Liu, W. & Pang, W. 2019. Are emotionally intelligent people more creative? A Meta-Analysis of the Emotional Intelligence–Creativity link. *Sustainability,* 11.

Zare, M. & Flinchbaugh, C. 2018. Voice, creativity, and big five personality traits: A meta-analysis. *Human Performance,* 32**,** 30-51.

Zehetner, A. & Zehetner, D. 2019. Complement or compensate? Interaction of IQ and Job Experience with Emotional Intelligence in Marketing Exchanges. *Journal of Marketing Development and Competitiveness,* 13.
